# The impact of mobility costs on cooperation and welfare in spatial social dilemmas

**DOI:** 10.1038/s41598-024-60806-z

**Published:** 2024-05-08

**Authors:** Jacques Bara, Fernando P. Santos, Paolo Turrini

**Affiliations:** 1 Department of Mathematics, University of Warwick, Coventry, CV4 7AL UK; 2 Informatics Institute, University of Amsterdam, 1098 XH Amsterdam, The Netherlands; 3 Department of Computer Science, University of Warwick, Coventry, CV4 7EZ UK

**Keywords:** Computational science, Nonlinear phenomena, Social evolution

## Abstract

From over-exploitation of resources to urban pollution, sustaining well-being requires solving social dilemmas of cooperation. Often such dilemmas are studied assuming that individuals occupy fixed positions in a network or lattice. In spatial settings, however, agents can move, and such movements involve costs. Here we investigate how mobility costs impact cooperation dynamics. To this end, we study cooperation dilemmas where individuals are located in a two-dimensional space and can be of two types: cooperators–or cleaners, who pay an individual cost to have a positive impact on their neighbours–and defectors–or polluters, free-riding on others’ effort to sustain a clean environment. Importantly, agents can pay a cost to move to a cleaner site. Both analytically and through agent-based simulations we find that, in general, introducing mobility costs increases pollution felt in the limit of fast movement (equivalently slow strategy revision). The effect on cooperation of increasing mobility costs is non-monotonic when mobility co-occurs with strategy revision. In such scenarios, low (yet non-zero) mobility costs minimise cooperation in low density environments; whereas high costs can promote cooperation even when a minority of agents initially defect. Finally, we find that heterogeneity in mobility cost affects the final distribution of strategies, leading to differences in who supports the burden of having a clean environment.

Increasingly we live in an urban world: four billion humans currently inhabit urban areas, which is projected to increase to two-thirds of the global population by 2050^1^. With this concentration of peoples, industries and activity comes an increase in pollution. Despite a global trend in fine particulate matter (PM2.5) reduction of around $0.2\%$ per year, 65% of urban areas showed an increase in PM2.5 levels and 71% of cities showed an increase in annual NO_2_ concentrations^2^. A study of 625 cities across China found decadal increases in PM2.5 that positively correlated with urbanisation^3^. As cities grow larger, some grow smarter, employing advanced sensing and monitoring systems^4^ in order to mitigate pollution and improve citizen health^5^. In China, for example, a nationwide policy programme dedicated to increasing public access to air quality information has been shown to trigger “cascading behavioural changes (...) that mitigated the mortality impact of pollution”^6^. The health benefits of this programme were estimated to outweigh its costs by almost a factor of 10: ¥93.3 billion in health benefits compared to ¥9.5 billion in costs, respectively. The increase in pollution - and knowledge thereof - has led to a reduction in habitants in more polluted destination cities^7^ and an increase in outflows from provincial counties, thus leading to depopulation^8^, which is largely driven by the residential movements of well-educated people at the beginning of their careers^8^ and skilled workers who show greater aversion to pollution than their unskilled counterparts^9^.

Urban pollution can result from both industries’ and citizens’ activities. The use of coal stoves in Ulaanbaatar (the capital of Mongolia) provides a clear example of the latter. In this city, due to a lack of municipal heating supplies, the use of raw coal stoves in *ger* households—as opposed to, for example, central heating in apartment blocks—accounts for roughly 80% of PM2.5 air pollution^10^. In particular *ger*, which is the Mongolian equivalent to the Turkic languages term *yurt*, are portable round dwellings constructed from a flexible and collapsible framework of light wood covered in insulating materials such as felt and canvas. Historically, they have been used extensively by nomadic cultures—due to their portability, affordability and high mobility—and *ger* households hold a large proportion of Ulaanbaatar’s population (in 2019, reportedly around 800,000 people^10^). Emigrating away from polluted areas may not be the only course of action; individuals may also perform environmentally friendly acts. In Germany, the large adoption of rooftop photovoltaic (PV) cells has been strongly attributed to highly localised imitation and technology diffusion^11^. In St. Joseph County, Indiana, where citizens are faced with the costly choice of upgrading private water systems (wells and septic tanks) to prevent contaminating other wells, researchers have proposed a theoretical framework to “understand how misaligned incentives can give rise to social dilemmas”^12^. Explicitly, their agents either paid a cost and reduced the risk of negatively impacting neighbours (cooperate) or paid nothing but risked contaminating others (defect).

As previous examples show, urban pollution—and ways to cope with it—suggests a spatial dilemma of cooperation, where agents cooperate, pollute, or move around, and whose decisions can have far-reaching consequences. Given such a dilemma, it is often hard to anticipate how policies affecting human mobility—here understood as residential mobility—affect cooperation concerning polluting decisions and citizens well-being. In this paper we investigate how imposing barriers to agents’ mobility, in the form of higher mobility costs, impacts cooperation in a spatial social dilemma of pollution. We develop analytic and agent-based models, inspired in methods typically used in social physics^13,14^ and evolutionary game theory^15–20^.

Spatial games typically occur on spatial lattices, as we consider here^15–17,21,22^. Some works have considered a continuous space, often resorting to dynamical systems approaches^23,24^ that treat cooperators and defectors as chemical-like species, tracking the *concentration* or fraction of cooperators/defectors rather than individuals. Others have considered complex networks^25–32^, where each node is an agent that plays a game with their neighbours. Similar models have been applied to management and policy-making. For instance, the $CO_2$ pollution stocks of all 28 EU member states (as of 2010) was modelled as evolving due to adjacency with other states and from investments (both from internal and external parties), in order to identify the impacts of different (inter)national policies^33^. Other works have had smaller scopes looking instead at pollution along river-networks in China, where nodes are locations on the river and edges indicate upstream-downstream and flow speed, either from the perspective of mechanism design^34^ or the fair allocation of clean-up costs^35^. Similarly to our approach, models of spatial social dilemmas were applied to private water systems^12^, assuming that agents exist on a lattice and spatial heterogeneity in the type of game being played (e.g. Prisoner’s Dilemma or Snowdrift) arises due to the orientation of houses and the underlying flow of groundwater. As previous works do, we assume that agents engage in spatial interactions; yet, we assume that they can move to sites where their payoffs are maximised, possibly paying a cost for that purpose.

In this paper, we consider a population of *N* agents that exist on a $L\times L$-sized periodic lattice playing a social dilemma of pollution (inspired in examples such as the mobility of *ger* households and the pollution due to coal stoves in Ulaanbaatar). Each agent, *a*, occupies a site in a grid-world. An agent is characterised by its position, $\varvec{r}_a$, and strategy, either Cooperate (*C*) or Defect (*D*) as denoted by $\sigma _a \in \{C,D\}$. Agents can move to unoccupied sites, as illustrated by Fig. 1. A cooperator pays a fee *f* to remove $\phi$ units of pollution from their site and all nearest neighbouring sites. A defector pollutes all sites a distance *r* away—1 unit for all sites within distance 1, while sites further away receive $r^{-2}$ units of pollution—up to a fixed distance *R*, in order to gain a benefit *g*. Unlike most other spatial social dilemmas^15–17,21–24,36–40^, therefore, our pollution game incorporates long-range effects. The strategy costs are fixed as $f=g=3.5$ such that when two agents are adjacent to one another and sufficiently far from all others, the pollution game is a social dilemma—by Supplementary Information (SI) Lemma 1 defection is dominant when $f+g>1+\phi$, while by SI Lemma 2 mutual cooperation of two adjacent agents is socially optimal when $f+g<2+2\phi$—for details regarding cost regimes, see the Two-player Social Dilemma Section in the Supplementary Information (SI). Agent based-simulations in Supplementary Fig S11 further indicate how defection is evolutionarily dominant in the limit of fast strategy updates, when all agents are fixed in locations.

Each agent *a* moves to the unoccupied site which, prior to movement, had the lowest level of pollution while paying a per-unit-distance mobility cost $\mu _a$. In other words, an agent moves to minimise the sum of its site’s pollution and the movement cost required to get there (see the movement objective function in Table 1). The total expense paid by an agent *a* is then the sum of the pollution of their current site $P(\varvec{r}_a)$, the strategy cost $\epsilon (\sigma _a)$ and the cost to move to $\varvec{r}_a$. An agent imitates a nearest neighbour with the highest payoff (or, conversely, lowest total expense), motivated by the strategy-update mechanism of highly-localised imitation found in the adoption of PV cells^11^, similarly to^36,37^. In other social dilemma works^19,38^, imitation is done instead using the pairwise comparison rule as the probability to imitate a neighbour. Computationally, our agent-based model progresses in discrete time steps comprising 4 phases where all agents perform the associated action in turn: I) imitation; II) movement; III) pollution; and IV) expense-calculation (for a full algorithm, see SI). Phase I occurs over a characteristic time-scale of $\tau _\sigma$ while phase II occur over a time-scale of $\tau _\mu$. In this work we look at two regimes: first, in the limit of fast movement and slow strategy revision, $\tau _\mu \ll \tau _\sigma$, in which phase I is skipped over; second, in a coevolutionary regime $\tau _\sigma = \tau _\mu = 1$ in which both phases happen with the same time-scale.

Notice that the computational time steps ($\tau _\sigma ,\tau _\mu$) used in our agent-based simulations are agnostic with respect to real temporal scales—in other words we are agnostic to how long 1 time step actually is (e.g. in seconds or days) –, and as such one can interpret our results for when time steps occur roughly on the scale of years, such that both imitation and movement are not as rapid as they may at first seem. For exceptionally mobile agents (e.g. vehicles or daily commutes) we can apply a similar methodology as our work but on much faster movement time-scales. For instance daily commute happens generally twice a day whereas residential mobility occurs once every few years potentially.

**Figure 1:**
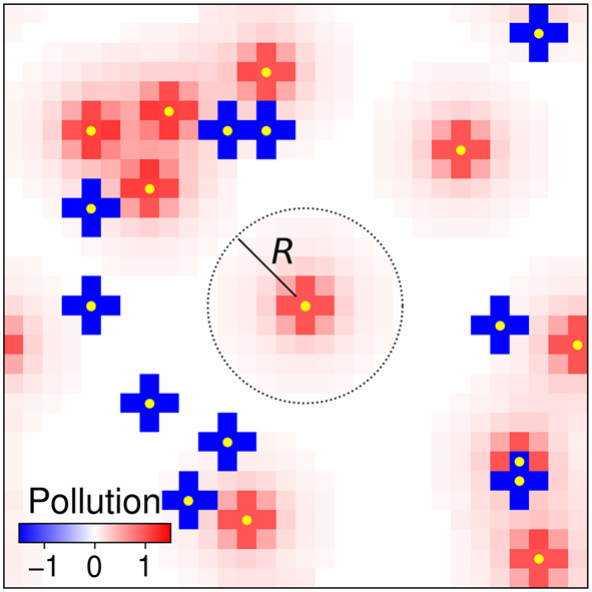
Agent-based model snapshot. Yellow circles indicate the locations of agents in the gridworld, either as defectors that produce pollution or as cooperators who remove it, where red (blue) squares indicate the level of positive (negative) pollution. As an example, the pollution cloud due to a defector in the centre of the lattice is illustrated by the dotted circle, which has radius *R*.

**Table 1:** Model summary for an agent *a* at site $\varvec{r}_a$ with strategy $\sigma _a$, having just moved from site $\varvec{r}'_a$.

Quantity	Cooperator $\sigma _a=C$	Defector $\sigma _a=D$
Strategy cost, $\varepsilon (\sigma _a)$	*f*	$-g$
Pollution, $P^{\sigma _a}(\varvec{r})$	$${\left\{ \begin{array}{ll} -\phi &{} \text { for } \Vert \varvec{r}_a,\varvec{r} \Vert \le 1\\ 0 &{} \text { otherwise.} \end{array}\right. }$$	$${\left\{ \begin{array}{ll} 1 &{} \text { for } \Vert \varvec{r}_a,\varvec{r} \Vert \le 1 \\ \Vert \varvec{r}_a,\varvec{r} \Vert ^{-2} &{} \text { for } 1 < \Vert \varvec{r}_a,\varvec{r} \Vert \le R \\ 0 &{} \text { otherwise.} \end{array}\right. }$$
Mobility cost	$\mu$	
Total movement cost	$\mu \Vert \varvec{r}_a,\varvec{r}'_a \Vert$	
Movement objective fn., $f(\varvec{r}_a,\varvec{r}_a')$	$P(\varvec{r}_a)+\mu \Vert \varvec{r}_a,\varvec{r}_a'\Vert$	
Expense, $E_a$	$P(\varvec{r}_a) + \varepsilon (\sigma _a) + \mu \Vert \varvec{r}_a,\varvec{r}'_a \Vert$	

Tabulated are the pollution/cleaning at site $\varvec{r}$ due to the agent $P^{\sigma _a}(\varvec{r})$, the strategy cost $\varepsilon (\sigma _a)$ and the mobility cost per unit distance, as well as the movement objective function $f(\varvec{r}_a,\varvec{r}'_a)$ and expense $E_a$.

Previous works considered the role of mobility on cooperation dilemmas. In particular, existing results show that random mobility can improve cooperation in the context of Prisoner’s Dilemmas played locally on a lattice^36,37^. Lee et al. noted that mobility costs can have a non-linear effect on cooperation in public goods games, when these are also played locally^38^.Our movement model deviates from previous works where cooperation dynamics are considered in spatial environments: it differs from the distance-limited model^32^, where agents only consider sites within a fixed distance of themselves though still move in order to minimise the pollution felt. It differs from random diffusion movement models^36,37^ where movement is entirely unbiased and undirected with respect to the alternative or target location; from success-driven movement^16^, where agents move to the site that would maximise payoff under fictitious play; from the directed migration model^24^, where cooperators are always attractive while defectors are always repulsive. Our model moreover differs even from other costly movement models, such as sophisticated cooperators^38^ that move only when their immediate neighbourhood contains a majority of defectors and the territorial raider model^39^, where agents have (overlapping) locations and may infringe upon other bordering territories.

The general framework of the latter was then used to tackle iterated public goods games from a stochastic perspective, where individuals can move from home locations to invade neighbouring locations via a costly Markov movement model^20^; increasing the cost was found to consistently increase staying propensities, but can impact cooperators differently than defectors depending on the relative speeds of mutation and movement. Later work^40^ found that the movement cost in the Markov model and the network topology (of home locations) mattered more than either the strategy update rule or even the average degree, long thought to be a critical aspect to the emergence of cooperation on networks^29^. In moving to deterministic cost-based movement, we can moreover analyse the effects of inequalities in mobility costs, motivated by the impacts seen previously^9,41^. In particular we consider a homogeneous case, where all agents pay the same mobility $\mu$, and a heterogeneous case, where there are two distinct groups: a fraction $\alpha <0.5$ of the population are in a minority group with mobility cost $\mu _m$ while the remaining $1-\alpha$ are in the majority group with $\mu _M$.

## Results

### Movement for small-*N* lattices

In order to build an intuition for the movement patterns that will emerge in heavily populated lattices we provide analytic results for the behaviour of small-*N* systems. In particular given $N\le 2$ agents, we identify the optimal sites for each agent to move into and discuss how this behaviour manifests and may present itself in larger systems. First, we begin with the single agent case in which the agent *a* is at the origin $\varvec{r}=(0,0)$, pondering where best to move. Due to the circular symmetry (up to spatial discretisation) we can consider the problem purely in the radial direction, that is we simply need to find the $r_*$ that globally minimises the movement objective function (see Table 1).

**Table 2:** Optimal location $r_*$ for a single agent at the origin to move to.

Agent	$\mu <2R^{-3}$	$2R^{-3}\le \mu <2$	$\mu >2$
Cooperator $r_*$	0	0	0
Defector $r_*$	*R*	$\root 3 \of {2/\mu }$	0

#### Single agent

Consider an isolated cooperator at the origin. It cleans all areas $\varvec{r}$ within a radius 1 of itself ($|\varvec{r}|\le 1$) by a factor $\phi \ge 0$ and thus creates a pollution field of $P(\varvec{r})=-\phi \mathbb {I}(|\varvec{r}|\le 1)$, where $\mathbb {I}$ is an indicator function that is 1 if the condition in its argument is satisfied and 0 otherwise. When considering where to move it must minimise its movement objective function $f(\varvec{r};\varvec{0}) = -\phi \mathbb {I}(|\varvec{r}|\le 1) + \mu |\varvec{r}|$ (see Table 1 for the general expression). However the objective function is minimised precisely at the origin $\varvec{r}=0$ since it monotonically grows with radial distance $r=|\varvec{r}|$. In other words, an isolated cooperator’s best movement strategy, therefore, is to remain stationary.

Consider instead an isolated defector at the origin. It has the following objective function (written in 1D radial form as there is circular symmetry),

1 $$\begin{aligned} f(r;\varvec{0}) = {\left\{ \begin{array}{ll} 1 + \mu r &{}\quad \text {for } r< 1 \\ r^{-2} + \mu r &{}\quad \text {for } 1\le r < R\\ \mu r &{}\quad \text {otherwise.} \end{array}\right. } \end{aligned}$$

which has three possible minima depending on the value of $\mu$ shown in Table 2. When $\mu$ is sufficiently small, the defector will always go to the site at the edge of its pollution cloud, however as $\mu$ increases (i.e. the cost of movement grows) the defector can only ever stay within its own cloud, until $\mu$ is so steep that it can only remain stationary.

#### Two agents

Consider now two agents $a_0$ and $a_1$ initially at sites $r_0=0$ and $r_1$, with strategies $\sigma _0$ and $\sigma _1$ respectively, with agent $a_1$ looking to relocate. To facilitate readability we illustrate the optimal movement for all combinations of strategies across a variety of initial distances $r_1$ in Supplementary Fig. S2. The distance moved by the mobile agent is monotonically non-increasing in mobility cost $\mu$, that is higher costs slow the agent down until eventually arresting it regardless of strategy at very high mobility costs $\mu =O(1)$. These high costs far outweigh any reduction in pollution, causing both cooperators and defectors to always remain stationary. The exact threshold at which this occurs depends both on the initial condition and the combination of strategies. For instance for two cooperators in most initial conditions, the optimal move is to remain stationary (see Lemma 4 in the SI for details), *except* when they share a single neighbouring site that benefits from both cleaning wells. In this case, the mobile agent moves to $r_*=1$ until the cost to move outweighs the pollution reduction at $\mu >\phi$.

In comparison, a mobile defector moves far more and over a wider range of cost-values, than their cooperator counterparts. When near a cooperator, the mobile defector $a_1$ mostly moves to the edge of the cooperator’s cleaning well that is furthest from $r_1$, in other words $r_* = -1$, in order to avoid their own pollution and to benefit from the cleaning well. With another defector, $a_1$ moves radially away from $a_0$ and itself opting for the edge of a pollution cloud where possible. Finally notice that when the stationary agent $a_0$ is a cooperator (blue and green lines), the mobile agent is always moves to within a distance 1 of the cooperator.

**Long-term Behaviour** Understanding the optimal movement in one time step allows us to understand long-term movement in some cases. In the single agent case since the mobility cost does not change over time, what the single agent does at one time step will be what they do for all time steps. That is, a cooperator will in the long-term always remain stationary while a defector may drift endlessly (moving either distance *R* or $\root 3 \of {2/\mu }$ each time) or remain stationary depending on the value of the mobility cost. The two-cooperator system $a_0$ and $a_1$ which at time *t* are separated by distance *r*(*t*), with initial condition $r(0)=r$, is similarly solvable. Two cooperators initially very near each other ($0<r\le 1$) will feel the effects of both cleaning wells, such that moving even infinitesimally far away will increase the total cost to move, and thus the two will remain stationary $r(\infty )=r$. If they are far away from one another ($r>2$) then they act as two separate single-cooperator systems and thus similarly remain stationary $r(\infty )=r$. Finally when they are near each other ($1<r\le 2$) and the mobility cost is sufficiently low ($\mu <\phi /(r-1)$) then it is beneficial to move closer in order once to feel both cleaning wells, otherwise again movement is too costly. In other words, for large-times, the two remain stationary for most values of *r* and $\mu$, otherwise if $1<r\le 2$ and $\mu <\phi /(r-1)$ then they move one step closer such that $r(\infty )=r-1$ (see SI for details).

### Simulating multi-agent systems

**Figure 2:**
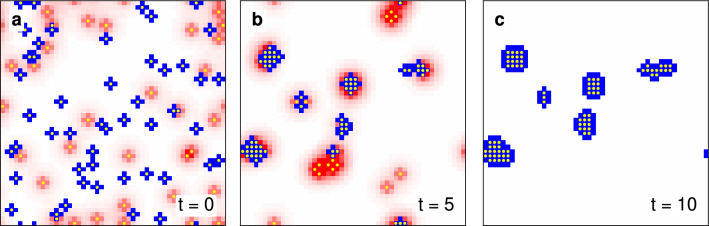
Snapshots of a single agent-based simulation. The lattice of size $L=50$ is shown at different times—(**a**) $t=0$, (**b**) $t=5$ and (**c**) $t=10$—with small yellow circles indicating the $N=80$ agents, that may move, imitate and cluster. Each site is coloured by the level of pollution: positive levels are in red, negative levels are in blue and sites with net zero in white. In particular colour-values are clipped such that the most red (blue) colours represent $\ge +2$ ($\le -2$) pollution, for illustration.

For a single agent-based simulation, Fig. 2 shows snapshots of a $L=50$ sized lattice—with pollution levels indicated by blue for negative values and red for positive values—occupied by $N=80$ agents. Initially agents are randomly distributed about the lattice (panel **a**) and over time begin to change strategies and to move. They do so by following the 4-phases of a time step: I) all agents change strategies, II) all agents move, III) all agents pollute and finally IV) all agents calculate their expenses. Even after 5 time steps (panel **b**) agents quickly cluster together as we can see clusters of cooperators (some with defectors in their periphery) and a few smaller clusters of defectors. The latter are the result of smaller cooperative clusters failing to grow sufficiently large, such that inbound defectors can easily convert them. In the long term $t=10$ (panel **c**), however, we see the remaining cooperative clusters cooperators have stabilised and moreover attracted defectors to ultimately convert them.

We observe the mechanisms by which either defection or cooperation can proliferate within a cluster and thus, potentially, the entire population: first, cooperators cluster together with some defectors in tow; second, either there are insufficient cooperators such that incoming defectors can easily infiltrate the cluster, or there are enough cooperators to be resilient against the change and thus convert incoming defectors. Next we investigate how changing mobility costs, population density and heterogeneity can modulate how difficult it is to form (large) cooperative clusters, and thus which of the two outcomes is more likely.

**Figure 3:**
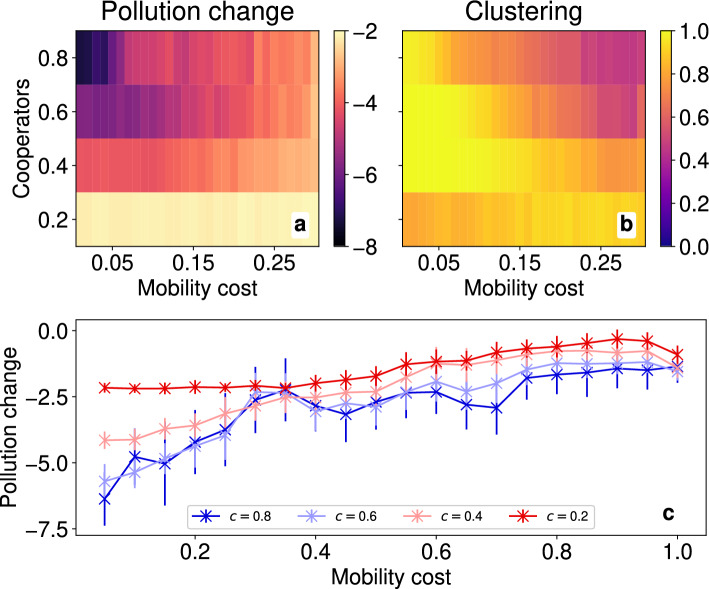
For a homogeneous population in the limit of fast movement, plots of the change in pollution and clustering. For lattices of size $L=50$ with $N=50$ inhabitants, (**a**) the change in PCP $\bar{\Delta }\hat{P}$ and (**b**) the clustering $\bar{\kappa }$ are displayed as heatmaps as we vary the cooperator fraction $c\in \{0.2,0.4,0.6,0.8\}$ and mobility cost $\mu \in \{0.01,0.02,\cdots ,0.3\}$. Brighter colours indicate smaller reductions in PCP in the left and higher levels in clustering on the right. The lineplot (**c**) moreover shows the change in PCP $\bar{\Delta }\hat{P}$ over a larger range of $\mu \in (0,1]$, with colour now denoting the fraction of cooperators. Vertical error bars in (**c**) represent 1 ensemble standard deviation. Note that for $c=0.2$, the errors are small but non-zero.

#### Limit of fast movement

We consider first the limit in which the characteristic time-scales of strategic behavioural changes $\tau _\sigma$ is far greater than the time-scales at which agents undergo residential movement $\tau _\mu$, $\tau _\sigma \gg \tau _\mu$. Moreover we simulate over the smaller time-scale, that is $T\sim O(\tau _\mu )$, such that strategies can be considered effectively fixed. In doing so for a homogeneous population (Fig. 3) of size $N=50$ we isolate the effects on clustering and reduction in pollution due to pure movement—for results on other population sizes and on correlations between PCP and clustering, see the Supplementary Information. In particular, lowering mobility costs $\mu$—or equivalently increasing mobility—increases both the mean clustering $\bar{\kappa }$ and the mean reduction in per-capita-pollution (PCP) $\bar{\Delta }\hat{P}$ (see Table 3 in Methods for the mathematical definitions). For lower costs, if some defectors found themselves in the middle of a cluster, the surrounding cooperators find it abundantly easy to move away and thus avoid encircling the defectors, who are at best left at the peripheries and borders of the cluster. When there are fewer cooperators, the initial cooperative clusters that form can be small and thus may not have enough space to accommodate all of the remaining defectors, such that the final $\kappa$ is lower. When agents are entirely free to move ($\mu =0$) cooperators can easily and optimally cluster together, hence the maximal $\bar{\kappa }$, forming typically a single large cluster (rather than many small ones). Reduction in PCP is dominated by the cores of these clusters, as central cooperators are maximally surrounded by agents whereas peripheral defectors waste some of their pollution on the unoccupied sites outside of a cluster. Finally, for even higher mobility costs ($\mu >0.3$), these trends continue in the same manner for both the pollution change (Fig. 3, panel **c**) and in clustering which we illustrate in Supplementary Fig. S5.

#### Coevolutionary regime

**Figure 4:**
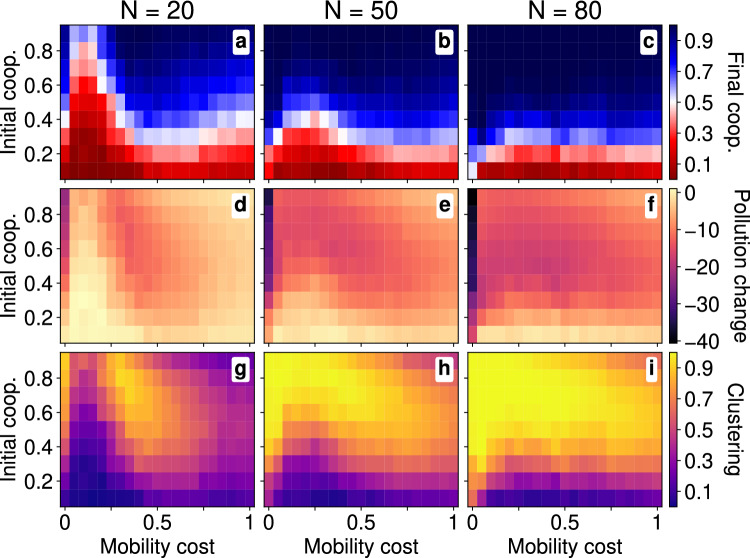
For a homogeneous population with imitation and movement, heatmaps of the final cooperation fraction, change in pollution and the clustering. In lattices of size $L=50$, we vary the mobility cost $\mu$ and initial cooperator fraction (initial coop.) *c*(0) while measuring: (top row, **a** - **c**) the final cooperator fraction $\bar{c}(T)$; (middle row, **d** - **f**) the pollution change $\bar{\Delta }\hat{P}$; and (bottom row, **g** - **i**) the clustering $\bar{\kappa }$. Moreover we do this for three population sizes: (left column, **a**, **d** & **g**) a few agents $N=20$; (middle column, **b**, **e** & **h**) a medium population $N=50$; and (right column, **c**, **f** & **i**) a large population $N=80$. Note that by small/medium/large population, we mean relative, effectively, to $L^2/\pi R^2\approx 32$ which is the minimum number of defectors for which the total pollution area equals the area of the lattice.

We now look at how the coevolutionary process proceeds *with* imitation as well as movement—in other words when $\tau _\sigma = \tau _\mu =1$—for a range of population sizes. In Fig. 4, on top of the reduction in PCP (middle row) and the clustering (bottom row) we see the final ensemble mean fraction of cooperators (top row). The blue regions in the top row show when cooperation proliferates $\bar{c}(T)>0.5$, even when a majority of the population are initially defectors $c(0)<0.3$ in the medium and dense lattices (middle and right panels). As a result of the increase in cooperators, there is a significantly higher reduction in PCP across most values of mobility cost and initial cooperation fraction, as shown by the purple regions in the middle row. Similarly the bright yellow regions in the bottom row show clustering increases in effectively the same way that PCP is reduced, in the same areas. In low density ($N=20$), however, cheap mobility ($\mu <0.4$) benefits defection more, causing a smaller reduction in PCP and very little clustering. For higher density or higher costs ($\mu >0.4$) the effects of mobility cost become far less pronounced instead the initialisation plays a much more important role. Finally, as in the fixed strategy case, free movement ($\mu =0$) always leads to heavy reduction in PCP and heavy increase in clustering, even though the fraction of cooperators is largely unchanged. In all, we find similarly to ref. ^32^ that mobility promotes cooperation in high density lattices as cooperators can more easily find one another and form large enough clusters; whereas in low density lattices mobility decreases cooperation since defectors can find cooperators more easily.

The non-linearity in mobility cost, particularly strong in the sparse lattice $N=20$ (left column), can be understood intuitively from the optimal movements of small *N*-lattices. For low, but non-zero costs ($0<\mu <0.4)$, cooperators are largely stationary while defectors will still move. As a result, the mobile defectors immediately move towards cooperators who, having remained static, have not clustered with any other cooperator yet. The cooperators *may* begin to move as defectors swarm around them and kick them out of stationarity. However, given the sheer number of defectors, the cooperators will more likely imitate their new defecting neighbours. Very quickly therefore defection proliferates before many (any) cooperative clusters form that are large enough to resist imitating defectors. Consequently the change in PCP is very poor as is the final level of clustering since most agents are now defectors.

At high mobility costs ($\mu >0.4$) however both cooperators and defectors are more likely to be stationary—as we have seen in the optimal movements of one or two agents—as any decrease in pollution is more than offset by the cost to move. Occasionally a defector may find a cooperator who then quickly moves in turn. As more single cooperators are made mobile, they can form clusters more quickly than defectors can swarm. This is evidenced by the high final cooperator fractions even when there are initially more defectors. Finally, when costs are completely non-existent ($\mu =0$) agents are no longer restricted to their localities, and instead have access to the entire lattice. As such cooperators will move, rather than remaining stationary, finding others and clustering, and can do so optimally. Defectors can equally find these cooperative clusters, however whether such clusters remain stable against defection becomes less a question of initial positions and more a question of how many cooperators are initially in the lattice.

#### Cost heterogeneity

**Figure 5:**
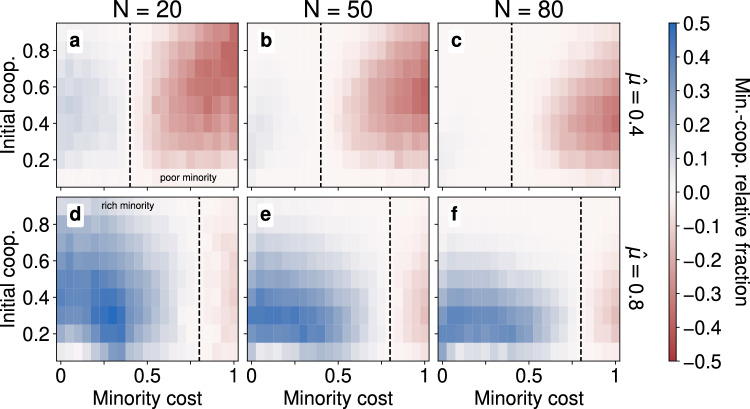
Interdependence of cooperation and group identity, heatmaps of the relative proportion of minority-cooperation against the population wide cooperative fraction. In lattices of size $L=50$, we vary the mobility cost $\mu$ and initial cooperator fraction (initial coop.) *c*(0). Negative values in red indicate a disproportionately smaller number of cooperators are in the minority group, a value of 0 in white gives exact independence while a positive values in blue indicate a disproportionately high number of minority-cooperators. Dashed lines represent when both groups have the same cost, i.e. $\mu _m=\mu _M=\hat{\mu }$, the areas to the left of the line indicate a richer minority while areas to the right indicate a poorer minority. The top row of panels (**a** - **c**) show the relative proportions when the mean cost is $\hat{\mu }=0.4$ while the bottom row (**d** - **f**) has $\hat{\mu }=0.8$. Different columns of panels, finally, indicate the size of the population from $N=20$ in the left, $N=50$ in the middle and $N=80$ in the right.

Having established the behaviour in homogeneous populations, we now shift our attention to heterogeneous populations where a fraction $\alpha =0.2$ of the population are in a minority group with mobility cost $\mu _m$ and the remainder are in a majority with cost $\mu _M$. We fix the mean mobility cost to either a low $\hat{\mu }=0.4$ or high value $\hat{\mu }=0.8$ and vary the minority cost $\mu _m$—and by extension the level of inequality. For brevity we leave the full results and figures on PCP reduction and clustering for both fast-movement limit and coevolutionary regime in the Supplementary Information (see in particular Supplementary Fig. S7–S9), while here we first summarise those results and focus on the relative fraction of cooperators in the coevolutionary regime (see Fig. 5).

In the limit of fast movement (see Supplementary Fig. S7), the level of inequality has less stark of an effect than the homogeneous case; the clustering and PCP are dominated instead by the mean cost. For a low mean cost $\hat{\mu }=0.4$ PCP reduction is larger than with a high mean cost $\hat{\mu }=0.8$, while the clustering on the whole is larger for the low mean cost. In the co-evolutionary regime (see Supplementary Fig. S8 and S9) we see that the effects of initial cooperation fraction *c*(0) and the heterogeneity are structurally the same as the homogeneous population, regardless of mean mobility cost $\hat{\mu }$. When the minority cost is low $\mu _m<0.3$ a similar defective spike to the one in Fig. 4—alongside the associated spikes in poor PCP reduction and low clustering—forms although for a far smaller set of initial cooperator fractions. Across all densities and mean cost, for a sufficiently high initial number of cooperators $c(0) > 0.4$ more than 40% of agents tend to cluster.

In order to better understand the effects of heterogeneity and costs on the minority-cooperator, Fig. 5 presents the minority-cooperator fraction *relative* to the global fraction of cooperators, that is $c_m\alpha ^{-1}-c\in [\alpha -1,1-\alpha ]$ where $\alpha N=0.2N$ is the size of the minority group. The figure shows in white when this value is 0 i.e. the cooperative fraction of the minority is equal to the global fraction such that there is independence between group-membership and strategic behaviour. On the other hand, when $c_m\alpha ^{-1}-c$ is positive (negative), shown in blue (red), there are a disproportionately high number of minority-cooperators (minority-defectors). The two distinct parameter regimes are separated by a dashed line ($\mu _m=\hat{\mu }$) and this separation emerges naturally out of the simulations as well. That is, when the minority are richer, $\mu _m < \hat{\mu }$, left of the dashed line, they disproportionately cooperate while when the minority are poorer, $\mu _m>\hat{\mu }$, right of the dashed line, they disproportionately defect. By the relationships between *c*, $c_m$ and $\alpha$ in Table 4 of the Methods, this equivalently implies that the majority group disproportionately cooperate. In other words, the group with the cheaper mobility cost always tend to disproportionately cooperate.

**Strategy-dependent mobility costs** In the previous section we looked at heterogeneous costs allocated by group—i.e. by a typology that is independent of their strategic action (cooperate or defect)—however this is not the only allocation possible. In particular we consider the scenario in which mobility costs are tied to agent strategies in the limit of fixed-strategies (fast movement), such cooperators have a cost $\mu _C$ and defectors a cost $\mu _D$. For fixed mean costs of $\hat{\mu }=0.4$ and 0.8 in lattices of population size $N\in \{20,50,80\}$, we find very non-linear behaviour in cooperator cost $\mu _C$ (for further detail see Supplementary Figure S10). As an illustrative example, the set of parameter values $\{\mu _C=0.1,c=0.8,\hat{\mu }=0.4\}$ (thus fixing $\mu _D=1.6$), corresponds to a lattice which contains $80\%$ cleaners who are entirely mobile while the remaining $20\%$ are polluters effectively acting as stationary, immobile factories. Moreover, as we only consider the limit of fixed-strategies for strategy-dependent costs, the analogy continues as factories do not typically overhaul themselves to become pollution cleaners. More generally, wholly tying mobility costs to strategies in a coevolutionary regime can inadvertently imply that mobility costs may rapidly change, which is somewhat unrealistic.

## Discussion

Residential mobility plays a key role in spatial pollution games and the emergence of cooperation. Even when the game parameters are fixed such that defection is dominant (individually optimal), when agents are mobile—that is subject to low mobility costs—cooperation can stabilise and proliferate, while per-capita pollution decreases. This occurs due to the emerging mechanism of *clustering*, where agents flock towards cooperators and form large groups. We show that in a two-player game, agents will move around and away from defectors for sufficiently low mobility costs while remaining stationary past a certain threshold cost, which depends on the combination of strategies. Moreover although there are no *a priori* differences in mobility between cooperators and defectors (i.e. they share the same cost to move), defectors effectively move more, even at larger costs. Despite the odds against cooperators however, especially in denser lattices cooperation becomes the majority strategy even when $70\%$ of the population is initially comprised of defectors. Simultaneously such lattices feel a large decrease in pollution as well as near-population wide clustering, even when costs are high.

In many previous works, mobility—across a variety of movement models—has been found to stabilise cooperative behaviour^15–17^ by allowing cooperators to cluster together. Other works reveal that even undirected random movement can enhance cooperation relative to the viscous (i.e. no-movement) case^36^ while strategy-heterogeneous movement (in the form of sophisticated agents) can improve cooperation in a majority of low-density cases^38^. Similarly, we find that in a cost-heterogeneous population a disproportionate fraction of cooperators emerges in the subgroup with lower costs (i.e. those agents who are more mobile). Different games (i.e. Prisoner’s Dilemma, Stag Hunt, etc.) are impacted to different extents by mobility, however regardless of the dilemma it does seem to enhance cooperation^37^. In reality, however, the dilemma a specific individual undergoes can be spatially-dependent^12^, and similarly in this work an individual’s game depends on the spatial distance to other players. Moreover density was found to have a critical effect on cooperation^32,38^; denser spaces allowed for clusters of cooperators to form more easily and quickly, which greatly stabilised cooperation in the population^32^. This clustering of cooperators occurs even when the underlying space is a dynamic network^42^ and stems from preferential attachment towards^19,43,44^ and unwillingness to detach from cooperators^27^, analogously to directed migration^24^. Most spatial social dilemma works, however, only consider the short-distance effects of defection. In this work, pollution effectively acts as a noisy signal of the cooperativeness of a region (as opposed to individuals) which can induce clustering as above, though exhibits a more complex relationship between movement, cooperation, density and clustering particularly in larger populations. In the homogeneous case we observe that, for low densities, small non-zero costs have led to almost no final clustering, due to the ease at which defectors found initial cooperative clusters; on the other hand for large densities these initial clusters grow sufficiently large to prevent defector saboteurs.

Heterogeneity in mobility costs, as a proxy for wealth/income inequality, has large consequences for the individual though seems to be less important at the population scale. When populations contain a rich-poor divide, that is one group pays less to move than the other group, individuals of the rich group (low mobility costs) are more prone to cooperate than the poor group as they can afford to shoulder the cost to cooperate. Not only can such individuals afford to cooperate and have polluting (thus costly) neighbours, but they can also afford to move into less polluted neighbourhoods which provide better environments for cooperation to emerge. At the population level, however, the level of cooperation, pollution and clustering are not as sensitive to changes to the minority group’s mobility cost, other than in a small range of intermediate costs. Instead the population mean cost plays a more important role, particularly increasing the mean cost causes fewer clusters to form and thus a smaller reduction in pollution.

Our model makes several implicit assumptions, that for transparency we acknowledge and that should be considered when using our model to make inferences about real-world case-studies. First, we assume a prior environment of no pollution and a space that is entirely undifferentiated—“*terra nullius*” in the words of ref. ^45^—which is arguably unrealistic as no environment is *a priori* empty: geographical features may hinder habitation, societal values may restrict which sites are available or there may be prior inhabitants. Second, our agents show no regard for the land or pollution *per se* and only care about it in so far as it is inconvenient/unpleasant for their own individual selves. This is a worst-case assumption, in other words, as we show that cooperation can emerge even when agents show no prior pro-environmental sentiment which would have made cooperation more attractive or feasible. Third, we have assumed that the only sources of (and consequently, the only responsibilities for) pollution are individuals. In reality, in the modern world pollution comes disproportionately from Global North institutions, be they governments or corporations^46^, and focusing purely on the individual scale further adds to this misattribution of responsibility.

Given the scope of this work, we have not included other location-based factors such as economic opportunities, proximity to amenities etc. since we wanted to focus on the sole role of mobility, as a simple but feasible baseline. We expect that adding place-based attachment preferences would only exacerbate the effects of mobility costs that we already discuss. There are a vast multitude of factors that an individual uses to decide where to move to (economic opportunities, local community, proximity to amenities, etc.) which can cause not just spatial heterogeneity but also heterogeneity in individual preferences (i.e. how a person weighs and balances all those factors together, personally), which leads to an explosion in complexity. In this project we focused on how specifically mobility impacts pollution, not on pollution as a whole. That being said, spatial heterogeneity can be included in our framework—though we leave this as avenue for future work –, by considering a background level of pollution that may be spatially heterogeneous and temporally evolving, which is driven by external forces i.e. not the agents themselves. In this way, the model can artificially project many external factors into effectively a single valuation or attractiveness of a location, while being able to include multi-sector dynamics^47^ and the presence of, for example, factories as static agents that continuously pollute and are unswayed by cooperative neighbours.

## Methods

### Pollution game

A set of *N* agents $\mathscr {A}$ exist on a doubly-periodic square lattice of size *L*, $\mathbb {T}^2_L$. Each lattice site can only be occupied by at most a single agent and we denote by $\varvec{r}_a$ the position of agent $a\in \mathscr {A}$. Each agent *a* moreover has a strategy $\sigma _a$ which determines first, the strategy cost $\epsilon (\sigma _a)$ for *a* and second, the pollution/cleaning caused by *a* at some location $\varvec{r}$, $P^{\sigma _a}(\varvec{r})$. An agent can either: cooperate ($\sigma _a=C$), by paying a fee $\epsilon (C)=f$ they remove $\phi$ units of pollution from their site and the nearest neighbouring *sites*; or to defect ($\sigma _a=D$), in order to gain a benefit $\epsilon (D)=-g$ they pollute all sites $\varvec{r}$ within radius *R* an amount $P^D(\varvec{r})$, where $r=\Vert \varvec{r}, \varvec{r}_a\Vert$ is the toroidal distance from the agent. The total pollution at any site $\varvec{r}\in \mathbb {T}^2_L$ at time *t*, denoted by $P_t(\varvec{r})$ is simply the sum of contributions from all agents $P^{\sigma _a(t)}(\varvec{r})$ to the site $\varvec{r}$.

2 $$\begin{aligned} P_t(\varvec{r}) = \sum _{a\in \mathscr {A}}P^{\sigma _a(t)}(\varvec{r}) \end{aligned}$$

**Toroidal Distance** Consider a circle of circumference *L*, $S^1_L \equiv [0,L) / L\mathbb {Z}$; a *L*-periodic (2-) *torus* is the Cartesian product of two such circles $\mathbb {T}^2_L \equiv S^1_L \times S^1_L$. Consider two points on the torus $\varvec{p},\varvec{q}\in T^2_L$ with $\varvec{p}=(x_p,y_q)$ and $\varvec{q}=(x_q,y_q)$. The distance $\Vert \varvec{p},\varvec{q}\Vert$ between the two points will be given by the geodesic distance.

3 $$\begin{aligned} \Vert \varvec{p},\varvec{q}\Vert \equiv \sqrt{ \min \big (|x_p-x_q|,L-|x_p-x_q|\big )^2 + \min \big (|y_p-y_q|,L-|y_p-y_q|\big )^2} \end{aligned}$$

### Costly mobility

Regardless of strategy, agents will move in order to minimise the pollution at their site, subject to a movement-cost-per-unit-distance $\mu$, henceforth a *mobility cost*. Specifically, an agent $a\in \mathscr {A}$ at site $\varvec{r}_a(t)$ at time *t*, will move to the site $\varvec{r}$ at time $t+1$ if $\varvec{r}$ is the minimiser of the current pollution there $P_t(\varvec{r})$ plus the cost to move $\mu \Vert \varvec{r},\varvec{r}_a(t)\Vert$ from $\varvec{r}_a(t)$ to $\varvec{r}$.

4 $$\begin{aligned} \varvec{r}_a(t+1) = \mathop {\mathrm {arg\,min}}\limits _{\varvec{r}} \big [P_t(\varvec{r}) + \mu \Vert \varvec{r},\varvec{r}_a(t)\Vert \big ] \end{aligned}$$

**Homogeneous and Heterogeneous Costs** In this work we will be considering two situations: one in which the population is homogeneous and thus pays a single universal cost $\mu$ and one in which the population is split into two disjoint subpopulations. Specifically in the latter, $\mathscr {A}=\mathscr {A}_m\cup \mathscr {A}_M$ is formed of two disjoint subpopulations that pay different costs to move: a minority group $\mathscr {A}_m$ that pay $\mu _m$ and a majority group $\mathscr {A}_M$ that pay $\mu _M$. In particular we let the minority group form a fraction $\alpha$ of the population ($|\mathscr {A}_m|=\alpha N$) leaving the majority group with the remaining $1-\alpha$ fraction of the population.

**Table 3:** Definition and description of the lattice metrics evaluated at time *t*.

Metric	Definition	Description
*c*(*t*)	$\frac{1}{N}\sum _{a\in \mathscr {A}}\mathbb {I}[\sigma _a(t)=C]$	Fraction of cooperators
$\Delta \hat{P}(t)$	$\frac{1}{N}\sum _{a\in \mathscr {A}} P[\varvec{r}_a(t)]-P[\varvec{r}_a(0)]$	Change in pollution-per-capita
$\kappa (t)$	$\frac{1}{N} \sum _{a\in \mathscr {A}} \mathbb {I}[\mathscr {N}_a(t) \ne \varnothing ]$	Fraction of agents directly neighbouring at least one other agent
$c_m(t)$	$\frac{1}{N}\sum _{a\in \mathscr {A}_m}\mathbb {I}[\sigma _a(t) = C]$	Fraction of minority-cooperators, for heterogeneous populations

### Imitation

The expense of agent *a* at time *t*, $E_a(t)$, is given by the sum of their site’s pollution $P[\varvec{r}_a(t)]$, their strategy cost $\epsilon [\sigma _a(t)]$ and the movement cost $\mu \Vert \varvec{r}_a(t),\varvec{r}_a(t-1) \Vert$ where $\mu$ is the mobility cost.

5 $$\begin{aligned} E_a(t) = P\big [\varvec{r}_a(t)\big ] + \mu \Vert \varvec{r}_a(t),\varvec{r}_a(t-1)\Vert + \varepsilon \big [\sigma _a(t)\big ] \end{aligned}$$

Given such expenses an agent *a* will look to her immediate neighbours $\mathscr {N}_a(t) = \{a':\Vert \varvec{r}_{a'}(t),\varvec{r}_a(t)\Vert \le 1, a'\ne a\}$ and imitate the neighbour with the lowest total expense. In other words at $t+1$ the strategy $\sigma _a(t+1)$ of agent *a* is given by the following.

6 $$\begin{aligned} \sigma _a(t+1) = \sigma _b(t), \qquad b = \mathop {\mathrm {arg\,min}}\limits _{a'\in \mathscr {N}_{a}(t)}\big [E_{a'}(t)\big ] \end{aligned}$$

### Lattice metrics

Consider a lattice $\mathbb {T}_L^2$ of size *L* containing *N* agents $\mathscr {A}$ and let agent $a\in \mathscr {A}$ have position $\varvec{r}_a(t)$ at time *t*. We list below the definitions of the cooperator fraction *c*(*t*), the change in pollution-per-capita (PCP) $\Delta \hat{P}(t)$, the clustering of agents $\kappa (t)$ where $\mathbb {I}(\cdot )$ is an indicator function which is 1 if its argument is true and 0 otherwise, and $\mathscr {N}_a(t) = \{a':\Vert \varvec{r}_{a'}(t),\varvec{r}_a(t)\Vert \le 1, a'\ne a\}$ is the set of immediate neighbours of agent *a*. Moreover, when considering heterogeneous populations we also measure the minority-cooperator fraction, $c_m$, which can also be seen as the joint probability to be both a cooperator and part of the minority group $\mathbb {P}(\sigma _a=C,a\in \mathscr {A}_m)$. Note that equivalent fractions $c_M,d_m,d_M$ for majority-cooperators, minority- and majority-defectors respectively can be fully determined by knowing $\alpha$ and measuring only *c* and $c_m$ as can be seen from Table 4.

In general where a mean of a variable is not specifically defined, e.g. in Table 3, then notationally we denote a per-capita mean by a hat and an ensemble mean by a bar. Concretely, for a population of $N=|\mathscr {A}|$ agents, in an ensemble $\mathscr {S}_N$ of simulations $i\in \mathscr {S}_N$, for some measured quantity *q* we denote $\hat{q}$ the per-capita mean and $\bar{q}$ the ensemble-mean. In particular for $q_a$ the value of *q* for node $a\in \mathscr {A}$ and $q_i$ the lattice metric for instance $i\in \mathscr {S}_N$ then $\hat{q}$ and $\bar{q}$ are given below. Plots and figures of lattice metrics (see Fig. 3-5 as well as Supplementary Fig. S5–S9) formally represent their respective ensemble-mean.

**Table 4:** Frequency table indicating population fractions of strategy and subpopulation group.

	Minority	Majority	Total
Cooperator	$c_m$	$c_M=c-c_m$	*c*
Defector	$d_m=\alpha -c_m$	$d_M=1-\alpha -c+c_m$	$1-c$
Total	$\alpha$	$1-\alpha$	1

7 $$\begin{aligned} \hat{q} = \frac{1}{N}\sum _{a\in \mathscr {A}}q_a \end{aligned}$$

8 $$\begin{aligned} \bar{q} = \frac{1}{|\mathscr {S}_N|}\sum _{i\in \mathscr {S}_N}q_i \end{aligned}$$

### Computational methods

**General parameters** We simulated cities of fixed size $L=50$ with varying population numbers $N\in \{20,50,80\}$, for fixed environmental factors $R=5$ and $\phi =5$ lasting for $T=50$ time steps—since at each time step all agents update themselves, convergence happens typically within the first 20-40 time steps, as can be seen in Fig. 2—over multiple runs. Parameters of the social dilemma are set to be $f=g=3.5$.

**Limit of fast movement** In order to understand how pure mobility affects the distribution of agents on the lattice, we first build a baseline in which all agents have fixed strategies. In doing so we can understand the migratory patterns that form and whether these patterns reduce pollution. As this is a simple baseline meant to develop intuition and gain insights, we run only $|\mathscr {S}_N|=50$ simulations per set of parameter values and only include $N=50$; for other *N*-values we include the results in the appendix. Finally as the fraction of cooperators is fixed, we instead measure the change in PCP, $\Delta \hat{P} = \hat{P}(T)-\hat{P}(0)$ and the clustering $\kappa$.

**Coevolutionary regime** Allowing for imitation to coevolve with mobility, we now increase the resolution in initial cooperation fraction $c(0)\in \{0.1,0.2,\cdots ,0.9\}$ over a considerably higher number of simulations $|\mathscr {S}_N|=200$ for both homogeneous and heterogeneous populations. Moreover on top of the PCP and clustering, we now measure the final cooperation fraction *c* and the minority-cooperator fraction $c_m$.

**Homogeneous cost** In the homogeneous case for each simulation $i\in \mathscr {S}_N[c(0),\mu ]$, all agents share the same mobility cost $\mu$, which we vary in $\mu \in \{0,0.05,\cdots ,1\}$. We restrict ourselves to $\mu \le 1$ since, by the analysis in the Supplementary Information, even for two nearby agents a $\mu =O(1)$ is sufficient to make an agent stationary in most cases.

**Heterogeneous costs** To minimise the dimensions of the parameter space: we vary the minority cost $\mu _m \in \{0,0.05,\cdots ,1\}$ while fixing the per capita mean cost $\hat{\mu }\in \{0.4,0.8\}$ - such that the majority cost is $\mu _M = (5\hat{\mu }-\mu _m)/4$ - and the minority fraction $\alpha =0.2$. As such we consider ensemble simulations of the form $\mathscr {S}_N[c(0),\mu _m,\hat{\mu }]$.

### Supplementary Information

Supplementary Information.

## Data Availability

The datasets, code, figures and other supplementary materials generated and/or analysed during the current study are available in the GitHub repository, https://github.com/JBara97/Costly-Movement-Pollution-Game.
