# Supplementary material for: The impact of mobility costs on cooperation and welfare in spatial social dilemmas
Source: Sci Rep. 2024 May 8;14:10572. doi: 10.1038/s41598-024-60806-z (PMC11079039; doi:10.1038/s41598-024-60806-z)
Supplement: Supplementary file 1 — Supplementary Information. [file 41598_2024_60806_MOESM1_ESM.pdf]

Supplementary Information

Jacques Bara[1,*], Fernando P. Santos[2,+], and Paolo Turrini[3,+]

[1]Department of Mathematics, University of Warwick, Coventry CV4 7AL, United Kingdom
[2]Informatics Institute, University of Amsterdam, 1098 XH Amsterdam, The Netherlands
[3]Department of Computer Science, University of Warwick, Coventry CV4 7EZ, United Kingdom
[*]jack.bara@warwick.ac.uk
[+]These authors contributed equally to this work

ABSTRACT

Mathematical Preliminary

Classifying Points

In this section we classify points given a function f, in order to identify optimal migratory patterns. For a function $f : X \to \mathbb{R}$, if it is differentiable at some point $x \in X$ then we denote its derivative as $f'(x) \equiv \frac{df}{dx}$.

Notable Point For a function $f : X \to \mathbb{R}$, a point $x_0 \in X$ is a *notable point* of f if it is (at least) one of a minimum, a maximum, an inflection point, a stationary point or a discontinuity.

Local Stationary Point Consider a function $f : X \to \mathbb{R}$ which is differentiable in an interval $x \in U \subseteq X$. If there exists a point $x_0 \in U$ such that $f'(x_0) = 0$ then x_0 is considered a *(local) stationary point* of f. Moreover if f is twice differentiable at x_0, the stationary point can be further classified as a *(local) minimum* (for $f''(x_0) > 0$) or *(local) maximum* (for $f''(x_0) < 0$).

Piecewise-defined Consider a function $f : X \to \mathbb{R}$ which is piecewise-defined at $x_0 \in U \subseteq X$. In particular let $f(x < x_0) = f_-(x)$ and $f(x > x_0) = f_+(x)$ where $f_\pm(x)$ are continuous differentiable functions in $x \in U$ - with $f(x_0)$ being either $f_-(x_0)$ or $f_+(x_0)$. We classify x_0 into two special cases depending on the derivatives of $f_\pm(x)$ at x_0, namely: a *(piecewise) minimum* if $f'_-(x_0) < 0$ and $f'_+(x_0) > 0$; a *(piecewise) maximum* if $f'_-(x_0) > 0$ and $f'_+(x_0 < 0)$. Note in this treatment, we are ambivalent as to whether x_0 is contained in the interval of f_- or f_+.

Discontinuity Consider a function $f : X \to \mathbb{R}$ that is discontinuous at $x_0 \in X$, where $f(x < x_0) = f_-(x)$, $f(x > x_0) = f_+(x)$ and $f_\pm(x)$ are differentiable. If at the discontinuity the two constituent functions are both increasing or both decreasing, i.e. $\text{sgn}(f'_-(x_0)) = \text{sgn}(f'_+(x_0))$, then we describe the discontinuity as a *(discontinuous) trap* if $\text{sgn}(f_\pm(x_0)) = \text{sgn}\big(f_-(x_0) - f_+(x_0)\big)$.

Global Minimum/Maximum For a function $f : X \to \mathbb{R}$, a point x_0 is the *global minimum* if $f(x_0) \leq f(x) \forall x \in X$. Similarly x_0 is a *global maximum* if $f(x_0) \geq f(x) \forall x \in X$. Note that a global minimum is not necessarily a local/discontinuity minimum for a discontinuous f.

Game Theory

Here we outline specific game theory notions that are used throughout the manuscript.

Dominant Strategy The strategy which minimises expense of a player, regardless of their opponent's strategy.

Nash Equilibrium When all players play their dominant strategy, this forms a Nash equilibrium.

Social Optimum The set of strategies that minimise the sum total of expenses across all players.

Social Dilemma When the Nash equilibrium is not the social optimum, the game is a social dilemma.

Two-player Social Dilemma

Consider two agents a_0 and a_1 at a distance r from each other, playing the symmetric pollution game described in the main manuscript. Table S1 presents the expense of a_0 in such a two-player game given both strategies σ_0 and σ_1. For example if a_0 cooperates but a_1 defects, and the two are at a distance r from one another, then the expense of a_0 is $E_1 = f - \phi + \mathbb{I}(r \leq 1) +$

$\mathbb{I}(1 < r \leq R)r^{-2}$. If they are more specifically immediate neighbours ($r = 1$) then agent a_0 gets an expense of $E_0 = f - \phi + 1$. From the expense matrix we can calculate the conditions for which set of strategies are a Nash equilibrium or a social optimum, thus arriving at Lemmas 1 and 2.

E_0	$\sigma_1 = C$	$\sigma_1 = D$
$\sigma_0 = C$	$f - \phi - \mathbb{I}[r \leq 1]\phi$	$f - \phi + \mathbb{I}[r \leq 1] + \mathbb{I}[1 < r \leq R]r^{-2}$
$\sigma_0 = D$	$1 - g - \mathbb{I}[r \leq 1]\phi$	$1 - g + \mathbb{I}[r \leq 1] + \mathbb{I}[1 < r \leq R]r^{-2}$

Table S1. Expense matrix for the two player game. Each element indicates the expense E_0 of agent a_0, given their strategy σ_0 (rows) and the other agent's strategy strategy σ_1 (columns). Note the indicator function $\mathbb{I}[\cdot]$ which is 1 if its argument is true and 0 otherwise.

Lemma 1. *For the two-player game, cooperation is the dominant strategy if $f + g < 1 + \phi$. Defection is dominant if the inequality is reverse.*

Proof. For each column in Table S1, taking the difference in the two elements removes all terms dependent on r, leaving only $E_0(\sigma_0 = C) - E_0(\sigma_0 = D) = f + g - (1 + \phi)$. If this quantity is positive, cooperation (strictly) dominates defection, else if this quantity is negative then defection is dominant. □

Lemma 2. *For the two-player game, mutual cooperation CC is the social optimum iff $f + g < (1+\phi)(1 + \mathbb{I}[r \leq 1]) + r^{-2}\mathbb{I}[1 < r \leq R]$. Mutual defection DD is the social optimum iff the inequality is reversed.*

Proof. The total expense $E = E_0 + E_1$ of the two player game is simply the sum of the expense matrix in Table S1 and its transpose, which is shown in Table S2.

E	$\sigma_1 = C$	$\sigma_1 = D$
$\sigma_0 = C$	$2f - 2\phi(1 + \mathbb{I}(r \leq 1))$	$f - g + (1-\phi)(1 + \mathbb{I}[r \leq 1]) + \mathbb{I}[1 < r \leq R]r^{-2}$
$\sigma_0 = D$	$f - g + (1-\phi)(1 + \mathbb{I}[r \leq 1]) + \mathbb{I}[1 < r \leq R]r^{-2}$	$2 - 2g + 2\mathbb{I}[r \leq 1] + 2\mathbb{I}[1 < r \leq R]r^{-2}$

Table S2. Total expense matrix for the two player game. Each element indicates the total expense $E_0 + E_1$ given the set of strategies. For example the total expense for mutual cooperation is $E(CC) = 2f - 2\phi(1 + \mathbb{I}(r \leq 1))$

Comparing the total expense of different strategy profiles and noting that $E(CD) = E(DC)$ will then give us similar conditions as above, namely,

$$\begin{aligned} E(CC) - E(CD) &= f + g - (1+\phi)(1 + \mathbb{I}[r \leq 1]) - r^{-2}\mathbb{I}[1 < r \leq R] \\ E(DD) - E(CD) &= -f - g + (1+\phi)(1 + \mathbb{I}[r \leq 1]) + r^{-2}\mathbb{I}[1 < r \leq R] \\ &= -\big(E(CC) - E(CD)\big) \end{aligned} \tag{1}$$

from which we can further derive that $E(CC) - E(DD) = 2\big(E(CC) - E(CD)\big)$. Thus if Eq. 1 is negative, then $E(CC) < E(CD) < E(DD)$, in other words mutual cooperation is the social optimum. Otherwise if Eq. 1 is positive then $E(CC) > E(CD) > E(DD)$ i.e. mutual defection is the social optimum. □

Both Lemmas 1 and 2 contain conditions for the difference in strategy costs $\varepsilon(C) - \varepsilon(D) = f + g$ in terms of the cleaning done by a cooperator ϕ and the inter-agent distance. These inequalities can moreover be visualised as the phase diagram in Fig. S1, that shows the regimes for which mutual cooperation/defection are socially optimum or form a Nash equilibrium. In particular three phases are formed: mutual defection is best individually and socially in the red diagonally-hatched region; mutual cooperation is best individually and socially in the green X-hatched region; and a social dilemma where defection is best for the individual even though cooperation is best for society in the orange vertically-hatched region.

Game parameters for the simulations in the manuscript are set to be $f = g = 3.5$, $\phi = 5$ and $R = 5$. In other words two agents immediately next to each other, but isolated from any others, will face a social dilemma as defection is dominant *but* all strategy combinations are socially optimum, that is Eq. 1 is exactly 0. With reference to Fig. S1, this corresponds to the point $(1, 2 + \phi)$ on the boundary between the red and orange regions. If, however, the two agents are any further away $r > 1$ then mutual defection becomes socially optimum. This is noteworthy as the results in the manuscript show that even in an environment so hostile to cooperation, cooperation can and does emerge due to the mechanisms of movement and clustering.

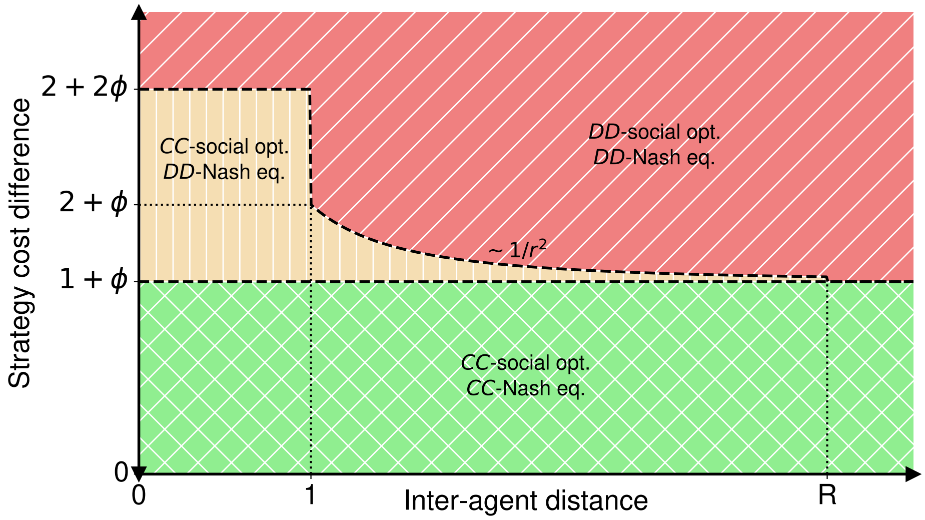

Figure S1. Phase diagram of the two-player pollution game for different inter-agent distances and strategy cost differences. Specifically, the x-axis indicate how far away the agents are from one another r, while the y-axis indicate the different in strategy costs $\varepsilon(C) - \varepsilon(D) = f + g$. Dashed black lines represent the phase boundaries given by Lemmas 1 and 2. Mutual defection (cooperation) is both social optimum and Nash equilibrium in the diagonnaly-hatched red region (X-hatched green region) while the vertically-hatched orange region shows a social dilemma in which mutual cooperation is socially optimum, whereas mutual defection is the Nash equilibrium. Dotted black lines indicate particular values of inter-agent distance and strategy cost difference.

Optimal Migration

Note that, since both the pollution and cost to move are piecewise-defined functions, total cost functions will in general be piecewise-defined, in other words will be comprised of several constituent functions which act on multiple disjoint subdomains. In this work we may occasionally leave the state of the boundary of two (or more, for the 2D problem) subdomains unspecified. In these cases we implicitly take the arrangement that minimises the cost function at the boundary. For instance in the 1D problem for a cost function $f : X \to \mathbb{R}$ if for some $x' \in X$, $f(x < x') = f_-(x)$ and $f(x > x') = f_+(x)$ then $f(x') = \min(f_-(x'), f_+(x'))$. Finally in order to deal with the periodic boundaries we limit ourselves to $|x| \leq L/2$ in the one-agent case and $\max(x', |x - x'|) \leq L/2$ where x' is the initial position of the mobile agent.

One Agent

Cooperator

Lemma 3. *A single isolated cooperator C remains stationary for any ϕ and any μ.*

Proof. Consider an isolated cooperator C w.l.o.g. at the origin. It cleans all areas $\boldsymbol{r}$ within a radius 1 of itself ($|\boldsymbol{r}| \leq 1$)by a factor $\phi \geq 0$ and thus creates a pollution field of $P(\boldsymbol{r}) = -\phi\mathbb{I}(|\boldsymbol{r}| \leq 1)$. When considering where to move it must minimise the objective function $f(\boldsymbol{r};\boldsymbol{0}) = -\phi\mathbb{I}(|\boldsymbol{r}| \leq 1) + \mu|\boldsymbol{r}|$. However the objective function is minimised precisely at the origin $\boldsymbol{r} = 0$ since it monotonically grows with radial distance $r = |\boldsymbol{r}|$. An isolated cooperator's best movement behaviour, therefore, is to remain stationary. □

Defector

Consider an isolated defector D w.l.o.g. at the origin. It has the following objective function (written in 1D radial form as there is circular symmetry),

$$f(r;\boldsymbol{0}) = \begin{cases} 1 + \mu r & \text{for } r < 1 \\ r^{-2} + \mu r & \text{for } 1 \leq r < R \\ \mu r & \text{otherwise.} \end{cases} \tag{2}$$

which has three possible minima depending on the value of μ. As such the defector will pick a random site at a radius r_* away from itself to migrate to. As we see from Table S3, when μ is sufficiently small, the agent will always go to the site at the edge of its pollution cloud, however as μ increases (i.e. the cost of movement grows) the defector can only ever stay within its own cloud, until μ is so steep that it can only remain stationary.

	$\mu < 2R^{-3}$	$2R^{-3} \leq \mu < 2$	$\mu > 2$
r_*	R	$\sqrt[3]{2/\mu}$	0

Table S3. For an isolated defector at the origin, the distance r_* of the site(s) that minimise the objective function Eq 2.

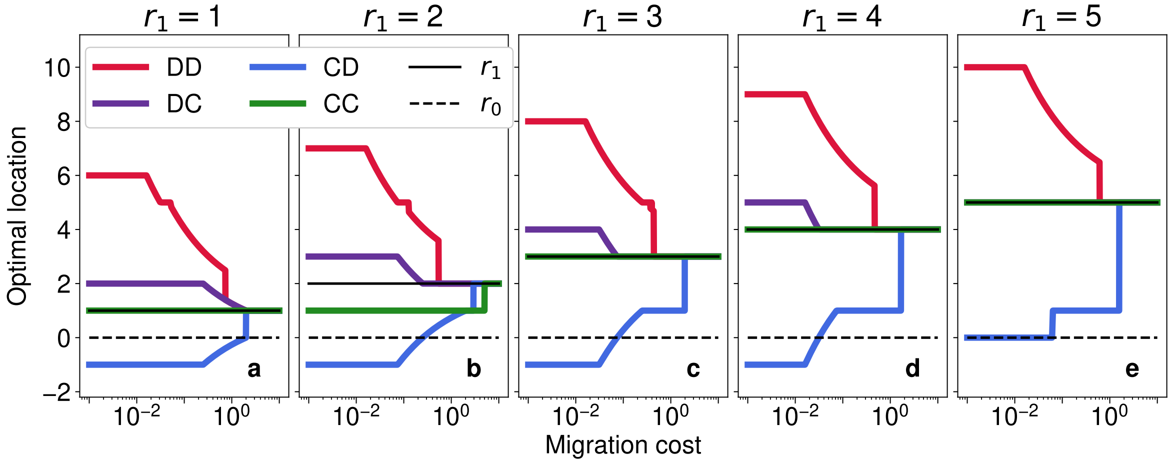

Figure S2. Optimal locations for the two-agent system. Agent a_0 is fixed at the origin, while different panels show various initial conditions r_1 for the mobile agent a_1. The locations r_0 (dashed black) and r_1 (bold black) are indicated. In each panel each (σ_0, σ_1) combination is represented by a different coloured line: two defectors (red); two cooperators (green); fixed cooperator with mobile defector (blue); fixed defector with mobile cooperator (purple). Note that in panel **e**, since the site at $r = 0$ is occupied, the optimal location for a_1 to move to would strictly be 0^-, that is the agent would move as close as possible to a_0 while remaining outside of their own poollution cloud. Other parameters: $R = 5$ and $\phi = 5$.

Two Agents

In this subsection we discuss for two adjacent agents, a_0 and a_1, initially at $\boldsymbol{r}_0 = (0,0)$ and $\boldsymbol{r}_1 = (r_1, 0)$, the optimal location to move $\boldsymbol{r}_*$ for agent a_1 as well as the other notable points in the system. Note that due to the circular symmetry we may restrict ourselves to the $1D$ problem in r (occasionally we switch notations to x). We first look at two cooperators for any value of r_1, finding their optimal next move and their long term behaviour. When there is at least one defector, we then focus only on adjacent agents $r_1 = 1$ for simplicity and tractability. Fig. S2 shows the global optimum for the two-agent movement objective function for 5 different initial conditions (panels in the figure) and combination of strategies (coloured lines in the figure).

Two Cooperators

A system of two cooperators very, a_0 and very near to each othera_1 at 0 and $r_1 \leq 1$ respectively is very simple. For $\mu > 0$ all costs are monotonically increasing away from the mobile cooperator. Just as in the single-cooperator therefore, for $\mu > 0$ there exists a unique global minimum at the initial position of the mobile neighbour. In other words a_1 remains stationary. For the far regime $r_1 > 2$, the two cooperator system behaves as two isolated bodies, as the two cleaning wells do not overlap. In other words the two cooperators remain stationary at all times.

On the other hand in the near regime $1 < r_1 \leq 2$, cooperator a_1 does have a global minimum not at a_1 depending on the value of μ. In particular, due to the cost function monotonically non-decreasing away from r_1 there are only two notable points, namely at $r_* = r_1$ – remaining stationary – and at $r_* = r_1 - 1$ – the edge of a_0's cleaning well closest to a_1. Finally given these two notable points we can simply evaluate the cost function f at $r_* \in \{r_1, r_1 - 1\}$ to find conditions on μ for the global minimum. Specifically, for $\mu > \frac{\phi}{r_1 - 1}$ then $r_* = r_1$ is a global minimum, thus a_1 remains static. Otherwise for $\mu < \frac{\phi}{r_1 - 1}$ then a_1 moves to neighbour a_0 by moving to $r_* = r_1 - 1$.

Having established the best response of agent a_1 given the initial conditions we can now reason about the long term behaviour of this system. More concretely, letting $r_\infty = \lim_{t \to \infty}(t)$ be the large-time limit of the inter-agent distance $r(t)$, we can derive r_∞ in terms of initial conditions.

Lemma 4. *Consider two cooperators ($\sigma_0 = \sigma_1 = C$) playing a pollution game, initially at a distance $r(0) = r$, subject to a mobility cost of μ with pollution parameter ϕ. If $1 \leq r \leq 2$ and $\mu < \frac{\phi}{r-1}$ then $r_\infty = r - 1$, otherwise $r_\infty = r$.*

Proof. Consider first the very near regime $0 < r_1 \leq 1$. At $t = 1$, the mobile agent a_1's best response is to remain stationary.

**Very near:** $r_1 \in (0,1]$		**Near:** $r_1 \in (1,2]$		**Far:** $r_1 \in (2,\infty)$	
r-Interval	$f(r)$	r-Interval	$f(r)$	r-Interval	$f(r)$
$(-\infty,-1)$	$\mu(r_1-r)$	$(-\infty,-1)$	$\mu(r_1-r)$	$(-\infty,-1)$	$\mu(r_1-r)$
$[-1,r_1-1)$	$\mu(r_1-r)-\phi$	$[-1,r_1-1)$	$\mu(r_1-r)-\phi$	$[-1,1]$	$\mu(r_1-r)-\phi$
$[r_1-1,r_1)$	$\mu(r_1-r)-2\phi$	$[r_1-1,1]$	$\mu(r_1-r)-2\phi$	$(1,r_1-1)$	$\mu(r_1-r)$
$[r_1,1]$	$\mu(r-r_1)-2\phi$	$(1,r_1]$	$\mu(r_1-r)-\phi$	$[r_1-1,r_1]$	$\mu(r_1-r)-\phi$
$(1,r_1+1]$	$\mu(r-r_1)-\phi$	$(r_1,r_1+1]$	$\mu(r-r_1)-\phi$	$(r_1,r_1+1]$	$\mu(r-r_1)-\phi$
(r_1+1,∞)	$\mu(r-r_1)$	(r_1+1,∞)	$\mu(r-r_1)$	(r_1+1,∞)	$\mu(r-r_1)$
**Optimal:** $r_*=r_1$		**Optimal:** if $\mu<\frac{\phi}{r_1-1}$ then $r_*=r_1-1$, otherwise $r_*=r_1$		**Optimal:** $r_*=r_1$	

Table S4. For a system of two cooperators a_0 and a_1 at 0 and r_1 respectively, the movement objective function for mobile a_1 is tabulated. Specifically there are three cases for how far away the two agents are from one another: (left two columns) the very near regime when $r_1 \in (0,1]$; (middle two columns) the near regime when $r_1 \in (1,2]$; and (right two columns) the far regime $r_1 \in (2,\infty)$. Moreover the optimal location r_* in all regimes are tabulated in the final row.

By symmetry, a_0's optimal movement would also be to remain stationary at $t=2$. Thus, neither will ever move i.e. $r_\infty = r$. Second, the far regime $r>2$ collapses to the two single-cooperator-case who individually remain stationary (Lemma 3). Hence in this regime $r_\infty = r$. Finally, in the near regime $1<r\le 2$, then there are two subcases. If $\mu > \frac{\phi}{r-1}$ then r (the initial position) is the global minimum for the movement cost, such that the cooperator remains stationary for all time so $r_\infty = r$, as seen previously. Otherwise if $\mu < \frac{\phi}{r-1}$ then the the global minimum is at $r-1$. The cooperator moves to this location and finds itself now within a new distance $r' = r-1$. Due to the symmetry regardless of whether a_0 or a_1 moves next, this new r' falls under the first case i.e. $0<r'\le 1$ and thus $r_\infty = r' = r-1$. □

Two Defectors

Consider two adjacent defectors D_0 and D_1 at $x=0$ and $x=1$ respectively, where D_1 wishes to move away. Again one can calculate the piecewise differentiable cost function $g(x)$. Unlike the one-defector-one-cooperator case in Section **??**, the two-defector case is symmetric (with respect to agent swap as well as to reflection around $x=0.5$), and as such we need only consider the perspective of one of the defectors.

$$g(x) = \begin{cases} \mu(1-x) & x<-R \\ \mu(1-x)+x^{-2} & -R\le x<-R+1 \\ \mu(1-x)+x^{-2}+(x-1)^{-2} & -R+1\le x\le -1 \\ \mu(1-x)+1+(x-1)^{-2} & -1<x\le 0 \\ \mu(1-x)+2 & 0<x\le 1 \\ \mu(1-x)+x^{-2}+1 & 1<x\le 2 \\ \mu(x-1)+x^{-2}+(x-1)^{-2} & 2<x\le R \\ \mu(x-1)+(x-1)^{-2} & R<x\le R+1 \\ \mu(x-1) & R+1<x \end{cases} \tag{3}$$

As seen in Figure S3 there are significantly more notable points with even more phases. Concentrating only on the global minimum, in other words the optimal agent choice, we see that there are 6 smooth phases. First, when μ is incredibly small ($\mu \le 2R^{-3}$) the edge of D_1's pollution cloud is the optimal place $x_* = R+1$ to move since the only cost is that of pure migration while she would feel 0 pollution. Second, as μ increases, it becomes worth it to stay within her own cloud, while remaining outside of her neighbours at $x_* = \sqrt[3]{2/\mu}+1$ when $2R^{-3} < \mu \le 2(R-1)^{-3}$. Third, for a while in $2(R-1)^{-3} < \mu \le 2(R-1)^{-3}+2R^{-3}$, D_1 remains at the edge of her neighbour's cloud in $x_* = R$. Fourth, for intermediate μ, the optimal distance is one of the real roots of a 6th order polynomial in $x_* = x_r(\mu)$, namely

$$\mu x_r^3(x_r-1)^3 - 2x_r^3 - 2(x_r-1)^3 = 0 \tag{4}$$

which can be found numerically. This stationary point minimum exists for $2(R-1)^{-3}+2R^{-3} < \mu \le 2.25$ however is only a global minimum until $\mu = \tilde{\mu}$ is the root of the following equation,

$$x_r\tilde{\mu} - 3\left(\frac{\tilde{\mu}}{2}\right)^{\frac{2}{3}} + x_r^{-2} + (x_r-1)^{-2} = 1 \tag{5}$$

which can be solved numerically. Fifth, for $\tilde{\mu} < \mu < 2$ the defector D_1 will increasingly remain closer to her initial position with $x_* = \sqrt[3]{2/\mu}$. Finally, for $\mu \geq 2$ the agent at $x = 1$ will remain stationary.

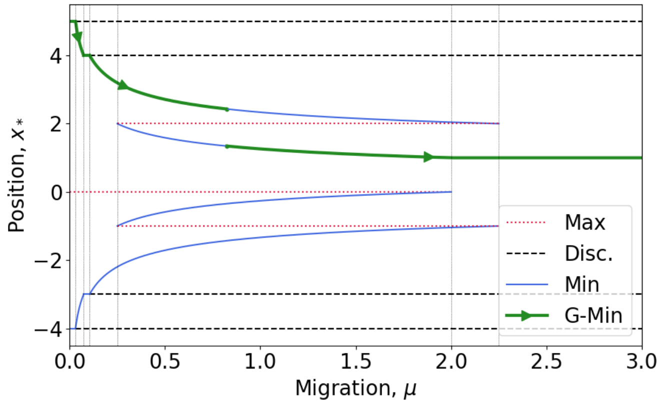

(a) All notable points of the cost function for two defectors.

Cost interval	x_*
$(0, 2R^{-3}]$	$R+1$
$(2R^{-3}, 2(R-1)^{-3}]$	$\sqrt[3]{2/\mu}+1$
$(2(R-1)^{-3}, 2(R-1)^{-3}+2R^{-3}]$	R
$(2(R-1)^{-3}+2R^{-3}, \tilde{\mu}]$	$x_r(\mu)$
$(\tilde{\mu}, 2)$	$\sqrt[3]{2/\mu}$
$[2, \infty)$	1

(b) Optimal location x_* for the mobile defector.

Figure S3. Illustration of notable points and explicit values for global minima for two adjacent defectors, for $R = 4$ and $\mu \geq 0$. The red dotted line represents maxima and solid lines minima of the function. In particular local minima are in blue while global minima - in other words the optimal site for the cooperator to move into - are in green. Finally dashed lines represent discontinuities that are neither minima nor maxima.

Defector and Cooperator

Consider a defector D at the origin and a cooperator neighbouring it, at $x = 1$. There are two cases to analyse, either the cooperator is mobile, or the defector is mobile. In both cases we will assume that the defector D is at the origin C

Mobile Cooperator Suppose, first, the cooperator is mobile, while the defector is fixed, then her migratory objective function $f_c(x)$, where x is the location of the site she considers moving into, is given below.

$$f_c(x) = \begin{cases} \mu(1-x) & x < -R \\ \mu(1-x) + x^{-2} & -R \leq x < -1 \\ \mu(1-x) + 1 & -1 \leq x < 0 \\ \mu(1-x) + 1 - \phi & 0 \leq x \leq 1 \\ \mu(x-1) + x^{-2} - \phi & 1 < x \leq 2 \\ \mu(x-1) + x^{-2} & 2 < x \leq R \\ \mu(x-1) & R < x \end{cases} \tag{6}$$

Note that when $\mu < 0$ - such that agents are always *paid* to move from their current location - the cooperator will always move away from the defector and in particular move infinitely far away, despite the existence of multiple local stationary points. However when $\mu \geq 0$ there are multiple notable points of various stability, as can be seen in Figure S4. Three notable points arise due to discontinuities in the objective function, namely $x_* \in \{-R, 2, R\}$ while the remainder arise from finding zero's of $f_c'(x)$.

From Figure S4 we moreover see 4 distinct phases across all notable points depending on the cost of migration μ, with phase changes occurring at $\mu \in \{2R^{-3}, 0.25, 2\}$ as shown by thin vertical dotted lines. However with respect to optimal agent behaviour, the cooperator has 3 distinct phases depending on μ.

First, if μ is very low then the cooperator always moves to the edge of her cleaning well, furthest from the defector, at $x_* = 2$. Second, as μ increases past $2R^{-3}$ the edge of her well becomes too expensive to occupy so she moves less and less $x_* = \sqrt[3]{2/\mu}$, until $\mu \geq 2$ when her best option is to remain completely stationary at $x_* = 1$. In discrete space the transition would not continuous nor smooth, in particular for $0.25 < \mu < 0.75$ she would always move to $x_* = 2$ while for $\mu > 0.75$ the cooperator remains stationary.

Mobile Defector For a mobile defector, the dynamics are largely the same as the mobile cooperator case, with all notable points being equivalent as can be seen in Figure S4. That is, except for the global minimum which discontinuously changes to $x_* = 0$ (i.e. for the defector to remain stationary) for a much smaller cost to move ($\mu = 2/3^{3/2} \approx 0.385$). In contrast the

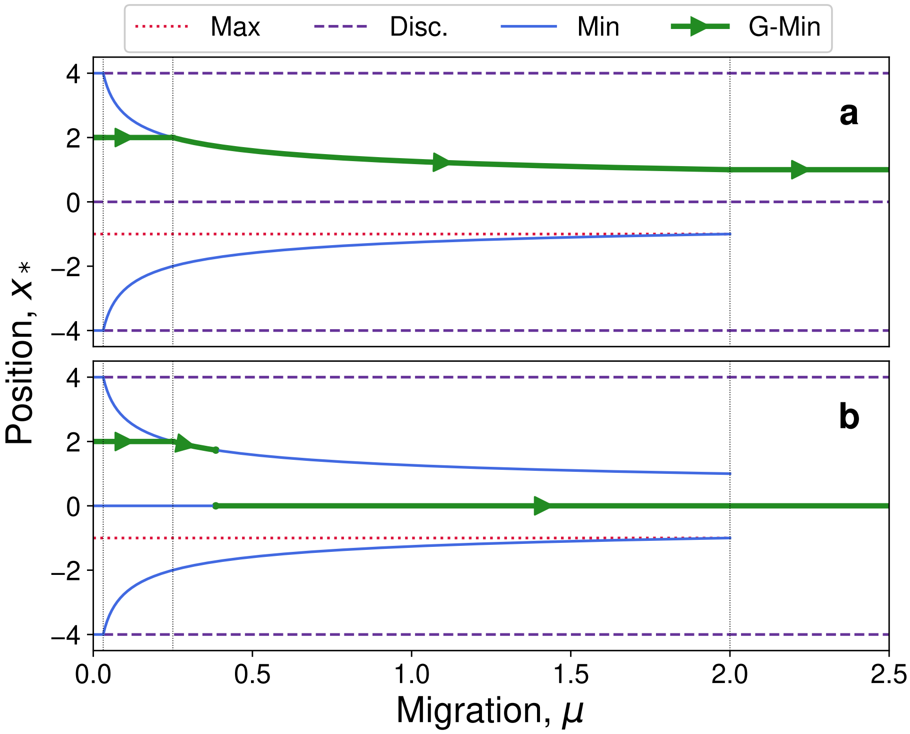

Figure S4. Notable points of the cost function for the one-cooperator-one-defector – Eq. (6) for the mobile cooperator (top) and Eq. (7) for the mobile defector (bottom) – for $R = 4$ and $\mu \geq 0$. The red dotted line represents a maximum (due to a discontinuity), while solid lines represent minima of the function. In particular local minima are in blue while global minima - in other words the optimal site for the cooperator to move into - are in green. Finally dashed lines represent discontinuities that are neither minima nor maxima.

cooperator is mobile for a larger range of μ, in other words the defector - once it has found a cooperative neighbour - is in some sense lazier than their neighbour. For completeness the cost function for the mobile defector f_d is given below.

$$f_d(x) = \begin{cases} -\mu x & x < -R \\ -\mu x + x^{-2} & -R \leq x < -1 \\ -\mu x + 1 & -1 \leq x < 0 \\ \mu x + 1 - \phi & 0 \leq x \leq 1 \\ \mu x + x^{-2} - \phi & 1 < x \leq 2 \\ \mu x + x^{-2} & 2 < x \leq R \\ \mu x & R < x \end{cases} \tag{7}$$

Agent-Based Simulations

Computational Methods In Algorithm 1 we explicitly lay out the order of operations of the agent based model, particularly highlighting how each phase is performed. Given a lattice $\mathbb{T}_L^2$ of size L, a set of N agents $\mathscr{A}$, a set of associated mobility costs $\{\mu_a\}$ – in the homogeneous case all $\mu_a = \mu$ are the same, whereas in the heterogeneous case $\mu_a = \mu_m$ if a is in the minority and $\mu_a = \mu_M$ otherwise –, the set of initial strategies $\{\sigma_a(0)\}$ and game parameters (clouds size R, amount of pollution cleaned by a cooperator ϕ, the cooperative strategy cost f, the benefit gained by a defector g and the total number of timesteps T) the algorithm returns the set of strategies at the final step $\{\sigma_a(T)\}$, the set of final positions $\{\boldsymbol{r}_a(T)\}$ and the change in per-capita-pollution (PCP) $\hat{P}$. Notice during initialisation, unique initial positions for each are randomly sampled from the lattice while a random sequence of agents is generated.

Algorithm 1 Agent-Based Model

Require: Lattice $\mathbb{T}_L^2$, agent set $\mathscr{A}$, mobility costs $\{\mu_a : a \in \mathscr{A}\}$, initial strategies $\{\sigma_a(0) : a \in \mathscr{A}\}$, parameters R, ϕ, f, g, T
Ensure: Strategies $\{\sigma_a(T) : a \in \mathscr{A}\}$, positions $\{\boldsymbol{r}_a(T) : a \in \mathscr{A}\}$, change in PCP $\Delta\hat{P}$

Initialise time $t \leftarrow 0$
Randomise initial positions $\boldsymbol{r}_a(0), \forall a \in \mathscr{A}$
Randomise sequence of all agents $[a_n]_{n=0}^{N-1}$
Initialise pollution matrix $P_0 \leftarrow 0_{L,L}$
for $\boldsymbol{r}$ in $\mathbb{T}_L^2$ **do**
    $P_0(\boldsymbol{r}) \leftarrow \sum_{a\in[a_n]} P^{\sigma_a(0)}(\boldsymbol{r})$ ▷ All agents pollute initially
end for
for t in $\{0, \cdots, T-1\}$ **do**
    for a in $[a_n]$ **do** ▷ Phase I: Imitate best performing neighbour b
        Update strategy $\sigma_a(t+1) \leftarrow \sigma_b(t)$, $b = \arg\min_{a' \in \mathscr{N}_a(t)} \left[E_{a'}(t)\right]$
    end for
    for a in $[a_n]$ **do** ▷ Phase II: Move by minimising objective fn.
        Update position $\boldsymbol{r}_a(t+1) \leftarrow \arg\min_{\boldsymbol{r}\in\mathbb{T}_L^2} \left[P_t(\boldsymbol{r}) + \mu \|\boldsymbol{r}, \boldsymbol{r}_a(t)\|\right]$
    end for
    for $\boldsymbol{r}$ in $\mathbb{T}_L^2$ **do** ▷ Phase III: Pollute
        $P_{t+1}(\boldsymbol{r}) \leftarrow \sum_{a\in[a_n]} P^{\sigma_a(t)}(\boldsymbol{r})$
    end for
    for a in $[a_n]$ **do** ▷ Phase IV: Evaluate new expense
        Update expense $E_a(t+1) \leftarrow P\left[\boldsymbol{r}_a(t+1)\right] + \mu \|\boldsymbol{r}_a(t+1), \boldsymbol{r}_a(t)\| + \varepsilon\left[\sigma_a(t+1)\right]$
    end for
end for
$\Delta\hat{P} \leftarrow \sum_{a\in[a_n]} \left(P_T(\boldsymbol{r}_a(T)) - P_0(\boldsymbol{r}_a(0))\right)/N$ ▷ Calculate change in per-capita-pollution (PCP)
return $\{\sigma_a(T)\}$, $\{\boldsymbol{r}_a(T)\}$, $\Delta\hat{P}$

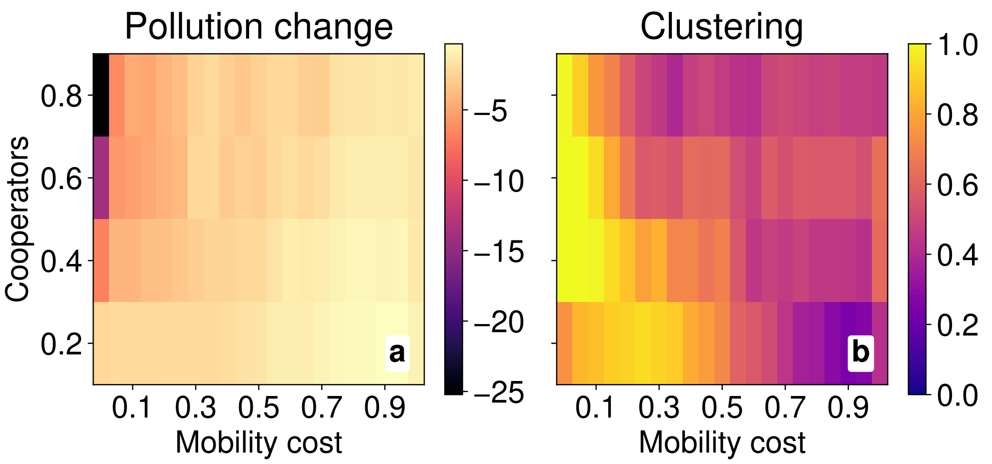

Figure S5. For a homogeneous population with fixed strategies, heatmaps of the change in pollution and clustering. For lattices of size $L = 50$ with $N = 50$ inhabitants, (**a**) the change in PCP $\bar{\Delta}\hat{P}$ and (**b**) the clustering $\bar{\kappa}$ are displayed as we vary the cooperator fraction c and mobility cost μ. Brighter colours indicate smaller reductions in PCP in the left and higher levels in clustering on the right.

Limit of Fast Movement for Various N

For completeness in the fixed strategy baselines, here we include results for populations of other sizes, namely $N \in \{20, 50, 80\}$, and introduce as an additional ensemble metric the Pearson correlation coefficient (PCC) between the reduction in PCP and the clustering. Additionally to the results of the main manuscript, Figures S6 and S10 show the statistical correlation as a Pearson coefficient between the clustering and the reduction in PCP $\rho(\Delta\hat{P}, \kappa)$ to identify when clustering plays an important role in PCP reduction. Namely, in the bulk of our parameter space the two quantities are negatively correlated (increasing clustering,

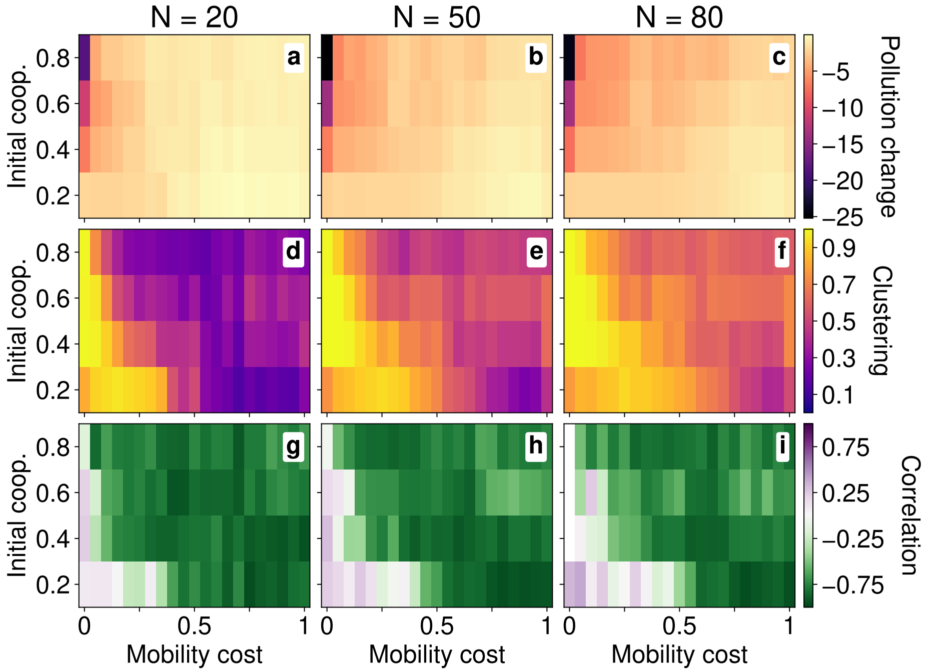

Figure S6. For a homogeneous population of varying sizes, heatmaps of the change in pollution, clustering and the correlation coefficient between the two. In lattices of size $L = 50$, we vary the mobility cost μ and number of cooperators c, measuring: (top row, **a** - **c**) the change in pollution $\bar{\Delta}\hat{P}$, (middle row, **d** - **f**) the clustering $\bar{\kappa}$ and (bottom row, **g** - **i**) the Pearson correlation between the two $\rho(\Delta\hat{P}, \kappa)$. In different columns moreover the number of agents is varied: (left column, **a**, **d**, **g**) $N = 20$, (middle column, **b**, **e**, **h**) $N = 50$, (left column, **c**, **f**, **i**) $N = 80$

decreases PCP). Where the correlation is very weak, however, there are either too few cooperators that PCP-reduction is heavily limited or that costs are so low that everyone can afford to join clusters regardless of how far they are.

Cost Heterogeneity by Group

For both the fast movement limit $\tau_\sigma \gg \tau_\mu$ (Fig. S7) and the coevolutionary regime $\tau_\sigma \approx \tau_\mu$ (Fig. S8-S9) we consider heterogeneous mobility costs, for which 20% of the population have a minority cost μ_m while the remainder have a majority cost μ_M. We fix the mean mobility cost to either a low value $\hat{\mu} = 0.4$ (left panels) or high value $\hat{\mu} = 0.8$ (right panels) and instead vary the minority cost μ_m – and by extension the level of inequality.

In the fast movement limit (Fig. S7), with the exception of a freely-moving minority ($\mu_m = 0$), the level of inequality has less stark of an effect than the homogeneous case. In particular both clustering and PCP are dominated instead by the mean cost; in both $\hat{\mu} = 0.4$ and 0.8 the overall behaviour due to varying c is qualitatively equivalent to the respective $\mu = 0.4$ and 0.8 for the homogeneous population (see Figure 3 in the main document). Moreover, as the majority cost is chosen to keep the mean cost fixed, the vertical slice $\mu_m = \hat{\mu}$ in both regimes and both metrics are exactly equal to the homogeneous case at the same value of μ. The biggest difference between low and high mean costs is the behaviour for low cooperation fractions ($c < 0.5$) in which increasing the minority cost improves (reduces) clustering in the low (high) mean cost regime.

For the coevolutionary regime in Fig. S8-S9 we see that the effects of initial cooperation fraction $c(0)$ and the heterogeneity, vis-à-vis the minority mobility cost, are structurally the same regardless of mean mobility cost $\hat{\mu}$ albeit to different intensity. For example the reduction in PCP is consistently maximised for intermediate values of $c(0)$, however in absolute terms the reduction when $\hat{\mu} = 0.4$ is consistently greater than the equivalent $\hat{\mu} = 0.8$. Similarly, clustering is maximised around $c(0) = 0.7$ regardless of both heterogeneity and mean mobility cost. When the minority cost is low $\mu_m < 0.3$ a similar defective spike to the homogeneous case – alongside the associated spikes in low PCP reduction and low clustering – arises though not as prominently nor for as large an initial cooperator fraction. Across all densities and mean cost, for a sufficiently high initial number of cooperators $c(0) > 0.4$ more than 40% of agents tend to cluster. Coupled with the previous results that lower costs tend to generally increase clustering, we see that the richer (i.e. lower mobility cost) subpopulation will cluster more than their counterparts. As such any cooperators within this subpopulation will be able to find one another more easily, and ultimately convert more defectors in their clusters. In turn this creates more cooperators and thus cooperation is favoured by the richer subpopulation. The most distinct effect, is on the fraction of minority-cooperators as shown in the second row.

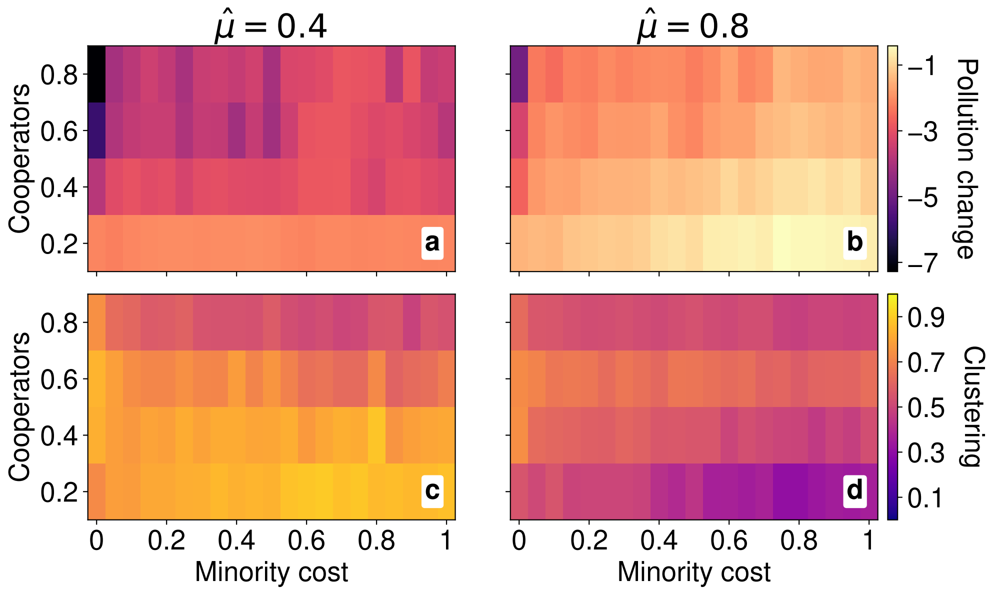

Figure S7. For a heterogeneous population in the limit of fast movement, heatmaps of the change in pollution and clustering. For lattices of size $L = 50$ with $N = 50$ inhabitants: (top row, **a** & **b**) the changes in pollution $\bar{\Delta}\hat{P}$ and (bottom row, **c** & **d**) the clustering $\bar{\kappa}$ are displayed as heatmaps, as we vary the cooperator fraction c and minority cost μ. The population mean cost is fixed at (left column, **a** & **c**) $\hat{\mu} = 0.4$ and (right column, **b** & **d**) $\hat{\mu} = 0.8$.

In medium and high density lattices most of the minority group become cooperators, as indicated by the light mint region, whereas in all cases when the initial fraction of cooperators is low $c(0) < 0.4$ most of the minority become defectors.

Cost Heterogeneity by Strategy

Moreover for the heterogeneous population we briefly consider the case when mobility cost is dependent on the strategy of an agent, that is cooperators have a cost μ_C and defectors have μ_D chosen so as to keep the mean cost again fixed to either $\hat{\mu} = 0.4$ or 0.8. For the sake of simplicity, we do not consider this allocation of costs when agents can change strategies, restricting it only to the fixed strategy case. In Fig. S10 we in particular see very nonlinear behaviour in the cooperator cost μ_C, across all densities. This presents an interesting and difficult avenue for research, due to the sheer complexity.

Post Peer-Review Response Discussions

Limit of Fast Strategy for Various N

Let us now consider the other extreme, when strategies are so quick to update that the agents are static. In this case, we use a more fine-grained range of population sizes $N \in \{10, 20, \cdots, 150\}$, for lattices of size $L = 50$, over 100 runs per parameter set. Since agents are static, imitation of an alternative strategy is massively conditioned upon whether or not the initial random location graces them with an immediate neighbour. For a fixed L, as N increases the likelihood to have an immediate neighbour similarly increases and thus the capacity – in effect, the total number of possible imitations – increases. As such, clustering is fairly minimal, at most the ensemble mean reaching 0.22, in other words 22% of agents had at least one immediate neighbour.

The restrictions due to the initial spatial distribution can be seen further by the fact that at most a net 5% of the population (not of the initial cooperators) have changed strategies. In particular the change is towards defection (denoted by redder regions in Fig. S11) – unsurprising given that defection is dominant – across the board, and does so by greater amounts as N increases. This is similarly reflected by the pollution change which typically increases, but by not very much at all – the most a single individual run increased by was only 1.38.

Effects of L in Homogeneous Populations

Since all agents have knowledge of the pollution of all sites in the lattice, L plays a very minor role in whether two agents will meet. That is even if they are 10 sites or 1000 sites away, in principle they *could* move to be adjacent to one another. Whether they actually do or not is unlikely for large distances given the movement model favours nearer sites, but it may be possible for very small costs or very low potential expenses. In order to verify this claim we repeat the homogeneous

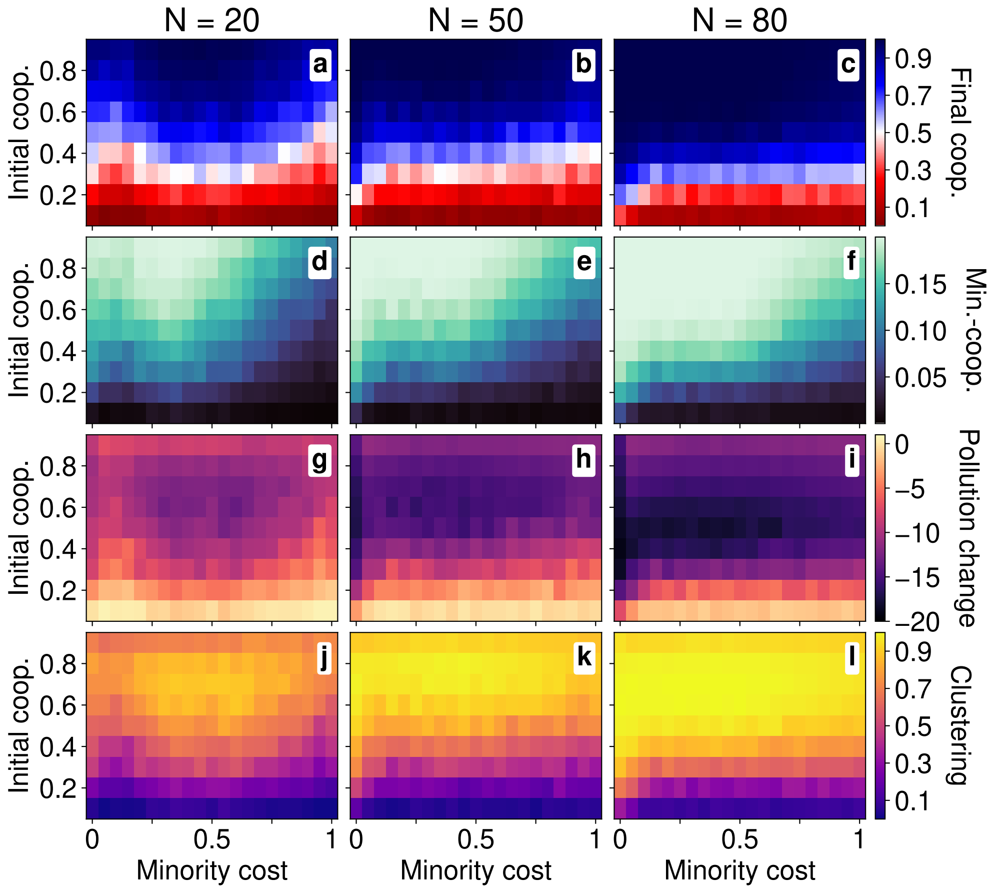

Figure S8. For a heterogeneous population with imitation and movement with low mean cost $\hat{\mu} = 0.4$, heatmaps of the final cooperation fraction, change in pollution and the clustering. We vary the mobility cost μ and initial cooperator fraction $c(0)$ while measuring: (top row, **a** - **c**) the final cooperator fraction $\bar{c}(T)$; (second row, **d** - **f**) the final minority-cooperator fraction $\bar{c}_m(T)$; (third row, **g** - **i**) the pollution change $\bar{\Delta}\hat{P}$; and (bottom row, **j** - **l**) the clustering $\bar{\kappa}$. Moreover we do this for three population sizes: (left column, **a**, **d** & **g**) a few agents $N = 20$; (middle column, **b**, **e** & **h**) a medium population $N = 50$; and (right column, **c**, **f** & **i**) a large population $N = 80$.

population experiments in the coevolutionary regime for $L = 100$ with $N = 50$ over the range $D = \{0.1N, 0.2N, \cdots, 0.9N\}$ and $\mu = 0, 0.1, \cdots, 1$, as shown by Fig. S12. We see that in comparison to the $L = 50$ results in the main text, the behaviour is qualitatively very similar with similar 'peaks' appearing for low (but non-zero) μ whereas for higher costs, the effects of the mobility cost becomes far less pronounced. A majority of agents cooperates by the end of the simulation, even when a majority of agents initially defected – again similarly to the original $L = 50$ result.

Even when R is considerably less than the lattice size L – such that it takes a significant number of, e.g., jumps to the left for a player to encounter their right-neighbour – the emergent behaviour of the system is qualitatively the same. In Fig. S13 we present the results for a homogeneous population of $N = L = 50$ agents with pollution clouds of radius $R = 2$. Again, we see the peak for low μ, the degenerate behaviour for free movement and the weakening impact of μ as μ increases.

Finally, we can use L in order to change the density of the lattice N/L^2. In the main text we have described $N = 80$ as the high density setting, which at first glance seems unreasonable given that $N/L^2 = 0.032$. However, as[1] found for the pollution game, one needs to account for the area that a defector impacts with their pollution, in effect πR^2 (the circular area of a cloud), such that the threshold between low and high density occurs at $N = L^2/(\pi R^2)$. To understand this, notice that an agent need not be immediately neighbouring another in order to feel their effects: an agent can be up to R away from a defector and still be impacted by their pollution. Thus in this work when we say "high density" we mean high relative to this value of $L^2/(\pi R^2)$.

To be certain, we have rerun the single homogeneous cost experiment instead with $N = 50$ and $L = 10$ such that the density is $N/L^2 = 0.5$, with the result presented in Fig. S14. In this setting, besides a difference between $\mu = 0$ and $\mu \neq 0$ (i.e. free and costly movement respectively) μ has very little effect on final cooperation fraction, change in PCP or clustering. In particular

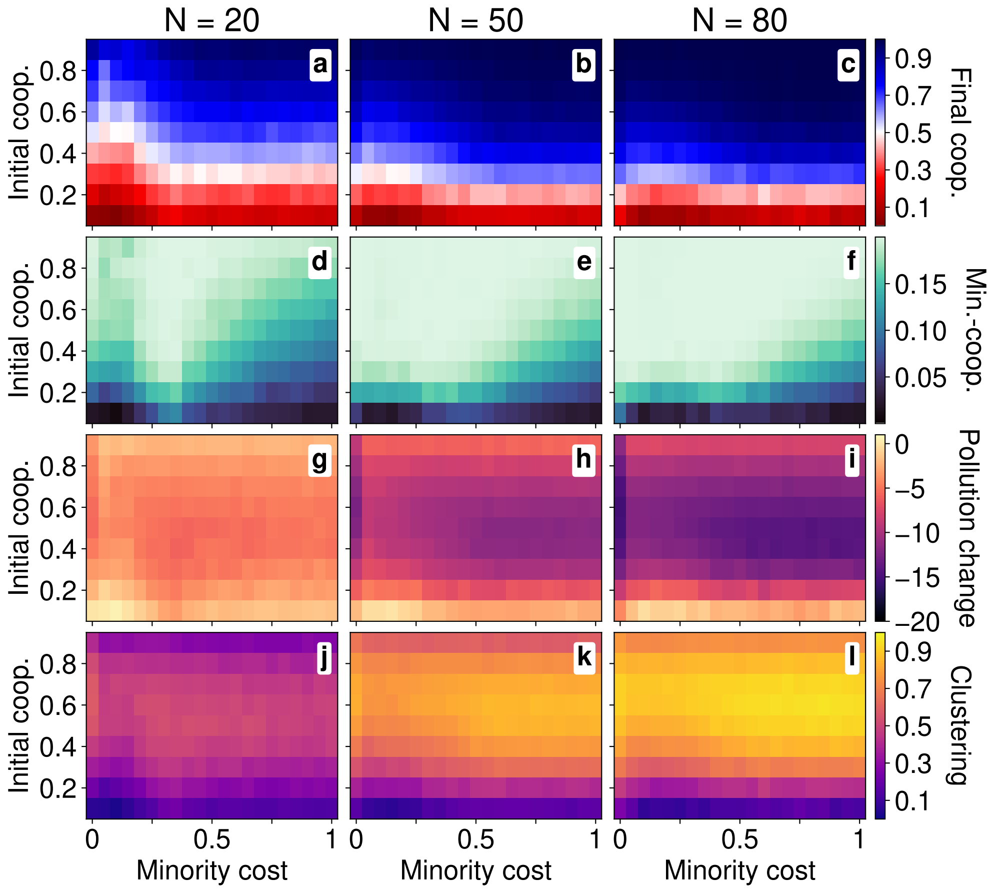

Figure S9. For a heterogeneous population with imitation and movement with low mean cost $\hat{\mu} = 0.8$, heatmaps of the final cooperation fraction, change in pollution and the clustering. We vary the mobility cost μ and initial cooperator fraction $c(0)$ while measuring: (top row, **a** - **c**) the final cooperator fraction $\bar{c}(T)$; (second row, **d** - **f**) the final minority-cooperator fraction $\bar{c}_m(T)$; (third row, **g** - **i**) the pollution change $\bar{\Delta}\hat{P}$; and (bottom row, **j** - **l**) the clustering $\bar{\kappa}$. Moreover we do this for three population sizes: (left column, **a**, **d** & **g**) a few agents $N = 20$; (middle column, **b**, **e** & **h**) a medium population $N = 50$; and (right column, **c**, **f** & **i**) a large population $N = 80$.

for the clustering the vast majority of runs led to a clustering $\kappa = 1$, leading to $\bar{\kappa} \approx 1$ for all choice of parameters. Even though half of the lattice sites are occupied, the entire lattice is initially affected by the pollution cloud of some defector. We see therefore the qualitative similarities with the $N = 80$ high density regime as presented in the main text.

References

1. Bara, J., Santos, F. P. & Turrini, P. The role of space, density and migration in social dilemmas. In *Proceedings of the 2023 International Conference on Autonomous Agents and Multiagent Systems*, AAMAS '23, 625633 (International Foundation for Autonomous Agents and Multiagent Systems, Richland, SC, 2023).

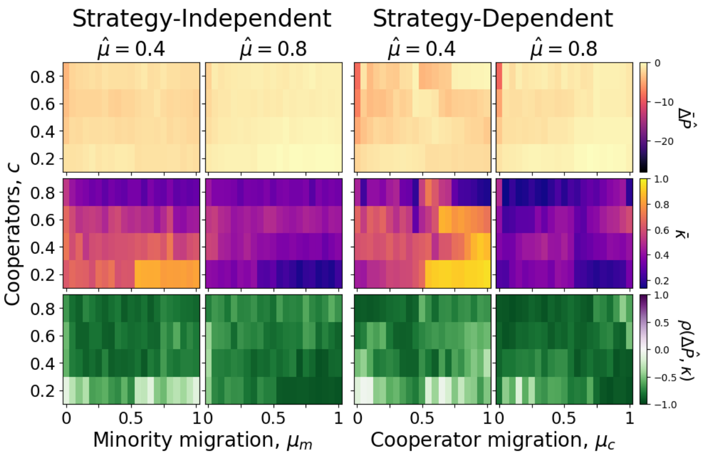

(a) $N = 20$

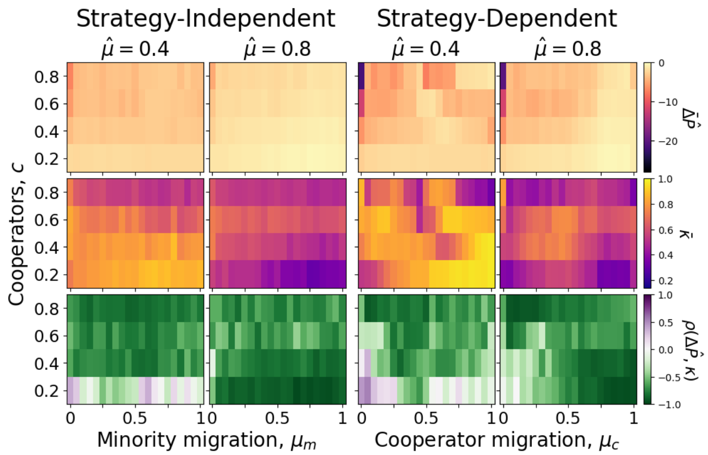

(b) $N = 50$

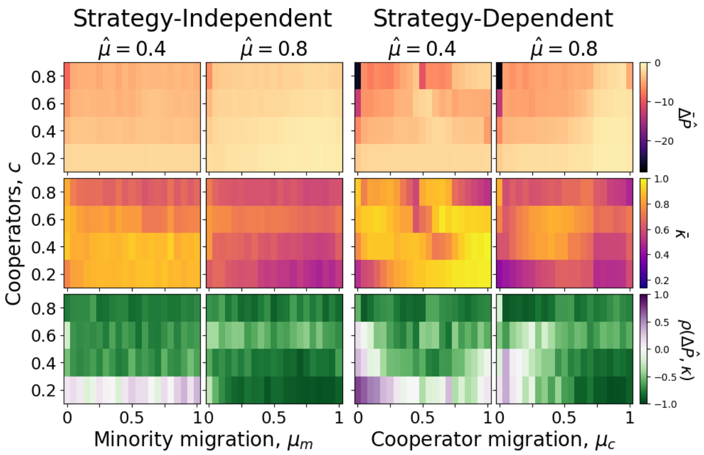

(c) $N = 80$

Figure S10. For a heterogeneous population of varying sizes, heatmaps of the change in pollution, clustering and the correlation coefficient between the two. Each subfigure Fig. S10a-S10c represent different values of N, the left hand side of which captures the strategy-independent cost allocation where 20% of the population are a minority, while the right hand side captures the strategy-dependent cost allocation where cooperators have different costs to defectors. The top row of panels indicate the change in pollution $\bar{\Delta}\hat{P}$, the middle row the clustering $\bar{\kappa}$ and the final row the Pearson correlation between the two $\rho(\Delta\hat{P}, \kappa)$.

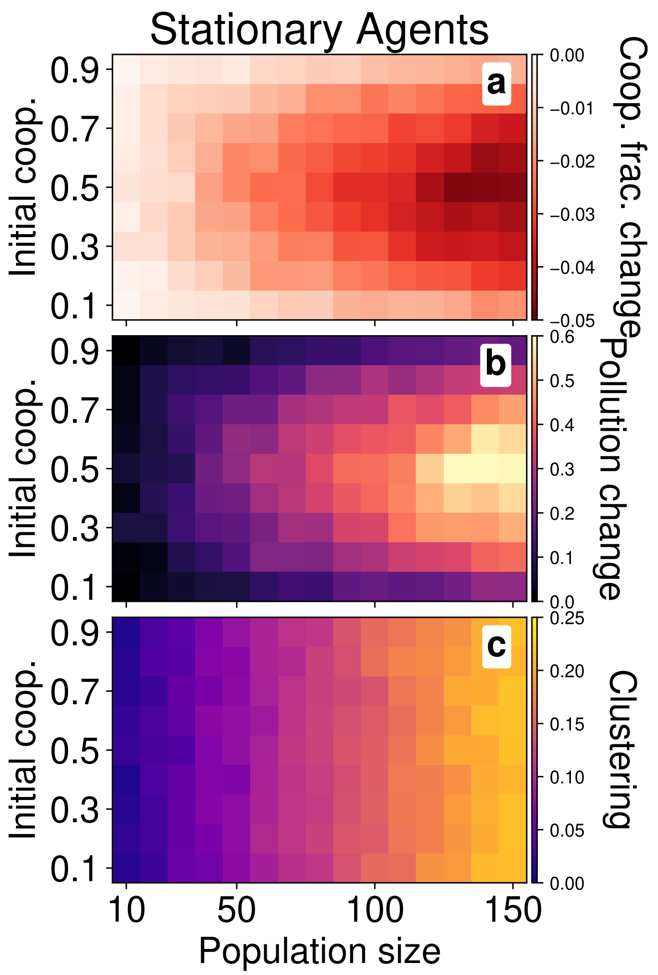

Figure S11. For a homogeneous but entirely static population with imitation, heatmaps of the final cooperation fraction, change in pollution and the clustering. In lattices of size $L = 50$, we vary the population size N (x-axis) and initial cooperator fraction $c(0)$ (y-axis) while measuring: (**a**) the change in cooperator fraction $\bar{c}(T) - c(0)$; (**b**) the pollution change $\bar{\Delta}\hat{P}$; and (**c**) the clustering $\bar{\kappa}$.

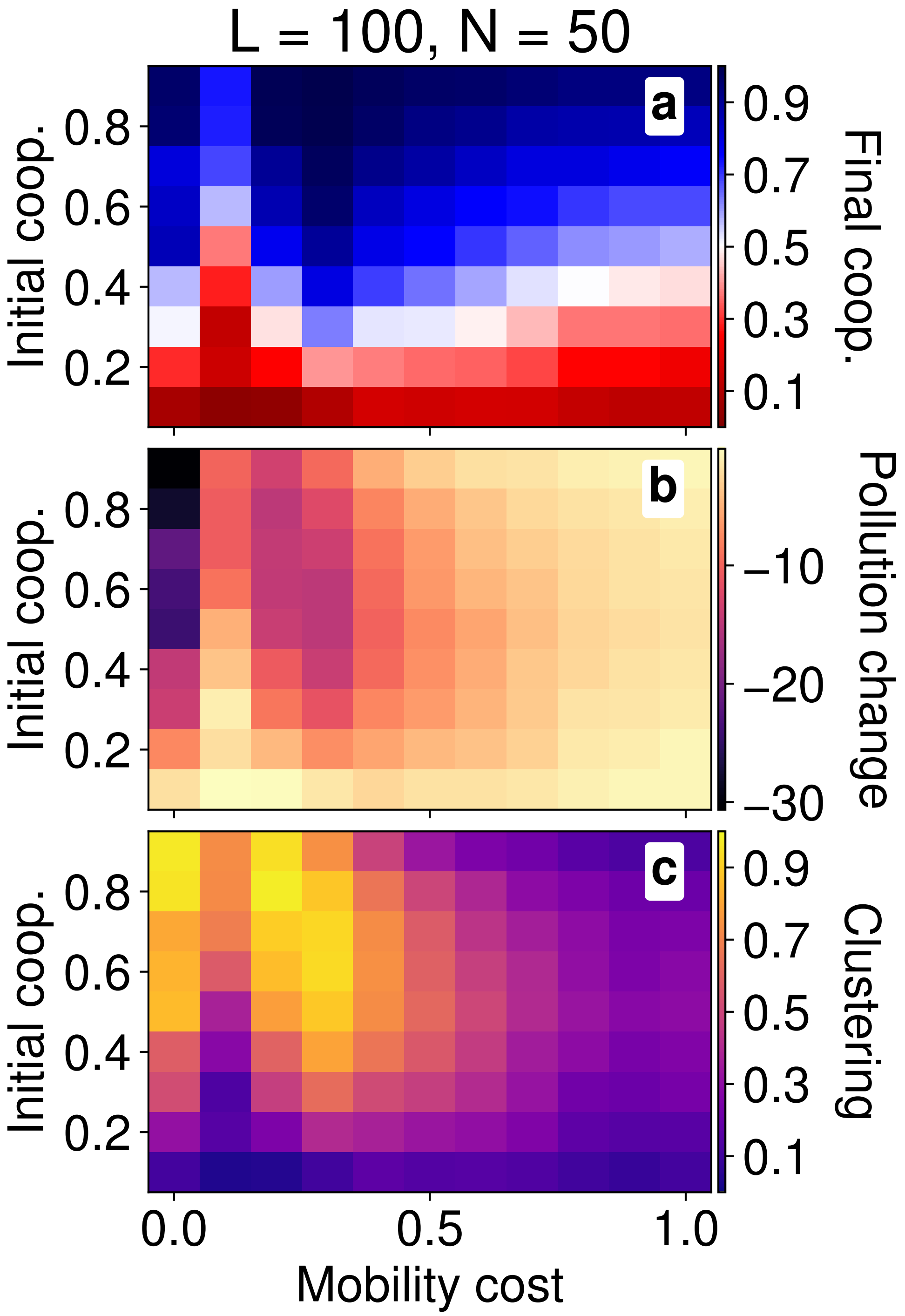

Figure S12. For a homogeneous population with imitation and movement, heatmaps of the final cooperation fraction, change in pollution and the clustering. In lattices of size $L = 100$, we vary the mobility cost μ (x-axis) and initial cooperator fraction $c(0)$ (y-axis) while measuring: (**a**) the final cooperator fraction $\bar{c}(T)$; (**b**) the pollution change $\bar{\Delta}\hat{P}$; and (**c**) the clustering $\bar{\kappa}$.

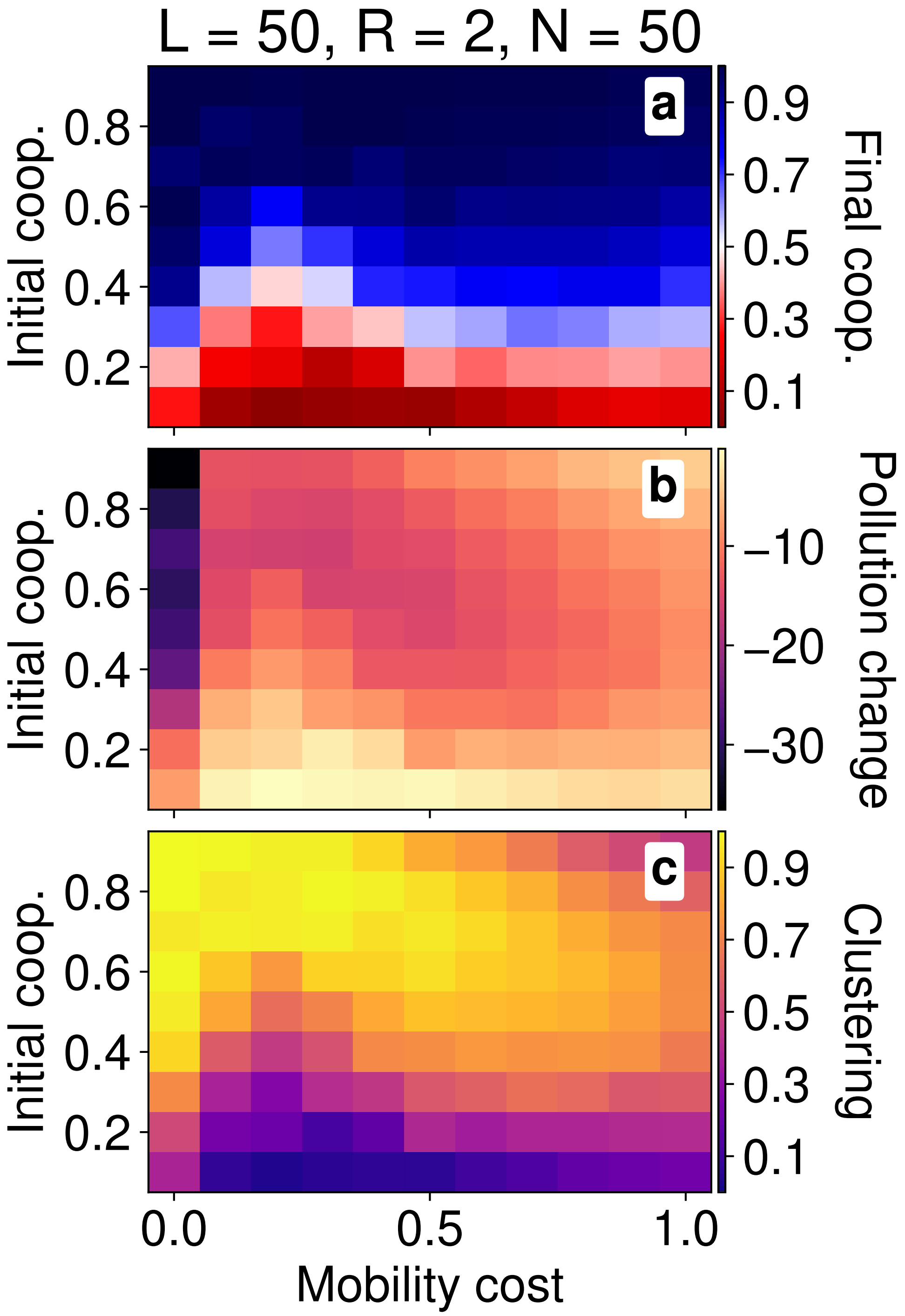

Figure S13. For a homogeneous population with imitation and movement, heatmaps of the final cooperation fraction, change in pollution and the clustering. In lattices of size $L = 50$, we vary the mobility cost μ (x-axis) and initial cooperator fraction $c(0)$ (y-axis) while measuring: (**a**) the final cooperator fraction $\bar{c}(T)$; (**b**) the pollution change $\bar{\Delta}\hat{P}$; and (**c**) the clustering $\bar{\kappa}$.

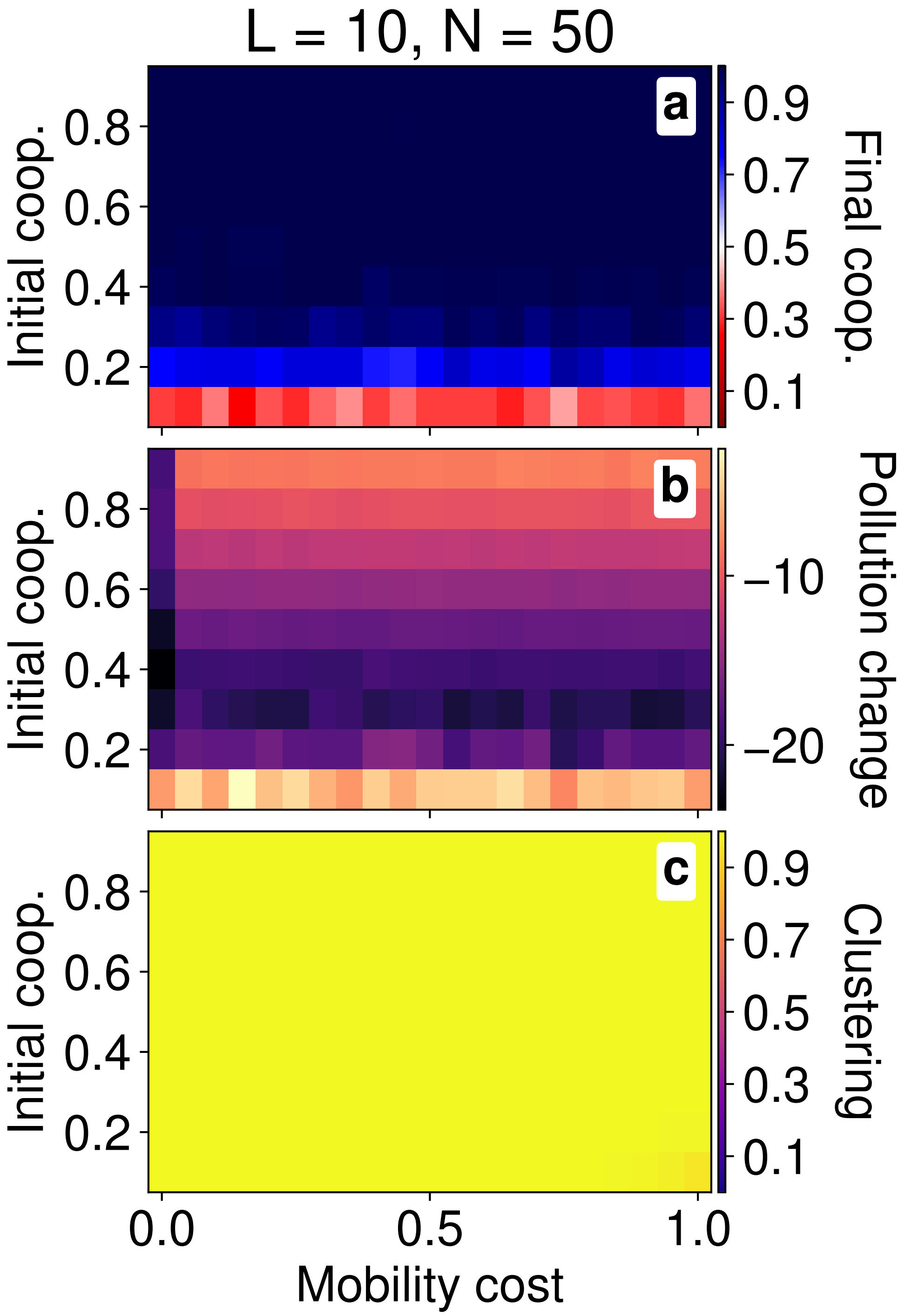

Figure S14. For a homogeneous population with imitation and movement, heatmaps of the final cooperation fraction, change in pollution and the clustering. In lattices of size $L = 10$, we vary the mobility cost μ (x-axis) and initial cooperator fraction $c(0)$ (y-axis) while measuring: (**a**) the final cooperator fraction $\bar{c}(T)$; (**b**) the pollution change $\bar{\Delta}\hat{P}$; and (**c**) the clustering $\bar{\kappa}$.
